# Risk factors for incident heart failure in age‐ and sex‐specific strata: a population‐based cohort using linked electronic health records

**DOI:** 10.1002/ejhf.1350

**Published:** 2019-01-07

**Authors:** Alicia Uijl, Stefan Koudstaal, Kenan Direk, Spiros Denaxas, Rolf H. H. Groenwold, Amitava Banerjee, Arno W. Hoes, Harry Hemingway, Folkert W. Asselbergs

**Affiliations:** ^1^ Julius Center for Health Sciences and Primary Care University Medical Center Utrecht, Utrecht University Utrecht The Netherlands; ^2^ Farr Institute of Health Informatics Research Institute of Health Informatics, University College London London UK; ^3^ Department of Cardiology, Division Heart & Lungs University Medical Center Utrecht, Utrecht University Utrecht The Netherlands; ^4^ The National Institute for Health Research, Biomedical Research Centre University College London Hospitals NHS Foundation Trust London UK; ^5^ Institute of Cardiovascular Science Faculty of Population Health Sciences, University College London London UK

**Keywords:** Heart failure, Incidence, Risk factors, Population attributable risk, Electronic health records

## Abstract

**Aims:**

Several risk factors for incident heart failure (HF) have been previously identified, however large electronic health records (EHR) datasets may provide the opportunity to examine the consistency of risk factors across different subgroups from the general population.

**Methods and results:**

We used linked EHR data from 2000 to 2010 as part of the UK‐based CALIBER resource to select a cohort of 871 687 individuals 55 years or older and free of HF at baseline. The primary endpoint was the first record of HF from primary or secondary care. Cox proportional hazards analysis was used to estimate hazard ratios for associations between risk factors and incident HF, separately for men and women and by age category: 55–64, 65–74, and > 75 years. During 5.8 years of median follow‐up, a total of 47 987 incident HF cases were recorded. Age, social deprivation, smoking, sedentary lifestyle, diabetes, atrial fibrillation, chronic obstructive pulmonary disease, body mass index, haemoglobin, total white blood cell count and creatinine were associated with HF. Smoking, atrial fibrillation and diabetes showed stronger associations with incident HF in women compared to men.

**Conclusion:**

We confirmed associations of several risk factors with HF in this large population‐based cohort across age and sex subgroups. Mainly modifiable risk factors and comorbidities are strongly associated with incident HF, highlighting the importance of preventive strategies targeting such risk factors for HF.

## Introduction

Heart failure (HF) is one of the leading causes of morbidity and mortality and is one of the initial presentations of cardiovascular disease (CVD).^1^ The lifetime risk in individuals aged 55 years and older is about one in five and the 5‐year survival ranges from 20–50% after first diagnosis.^2^, ^3^, ^4^

In recent decades, several risk factors for developing HF have been established, such as high blood pressure (BP), diabetes, smoking, and obesity.^5^, ^6^, ^7^, ^8^ The contribution of these risk factors may differ substantially, considering the age and clinical presentation of CVDs differ greatly amongst men and women.^1^ Therefore, the associations of such risk factors with HF should be evaluated separately in men and women across a range of age groups. Furthermore, management of well‐known risk factors could be partly responsible for a declining incidence of HF.^9^ However, as ‘classic’ risk factors such as hypertension are successfully treated by BP‐lowering medication to decrease CVD risk, in a population where such strategies are implemented, the equilibrium between risk factors, dependent on age, sex and risk factor distribution, could have shifted, and relatively less known risk factors could emerge.

Previous studies of risk factors for HF may lack data richness or sheer volume for a thorough assessment of differences in the contribution of risk factors across different patient groups of interest (notably strata of age and sex).^10^, ^11^, ^12^ Very large databases of electronic health records (EHR) may provide the opportunity to study risk factors among age‐ and sex‐specific groups of patients in the general population.

In the current study, we studied a large population‐based cohort using EHR, with a highly heterogeneous HF phenotype, to identify risk factors for developing HF and to compare these risk factors between men and women across different age groups.^13^

## Methods

### Study population

A cohort of 871 687 individuals was constructed from the CALIBER resource (CArdiovascular research using LInked Bespoke studies and Electronic health Records), which links four sources of EHR in England: primary care records from the Clinical Practice Research Datalink (CPRD), secondary care hospital discharges in Hospital Episodes Statistics (HES), disease registration in the Myocardial Ischaemia National Audit Project (MINAP) registry and the national death registration in the Office for National Statistics (ONS) registry.^13^

Individuals were included if they were 55 years or older between 1 January 2000 and 25 March 2010, if they had been registered with a general practitioner for at least 1 year, in a practice that had at least 1 year of up‐to‐standard data recording in CPRD. The last date of the previously mentioned occasions was considered cohort entry date (index date).

We excluded individuals with a history of HF in CPRD, HES or MINAP before their index date. Individuals were censored at first diagnosis of HF, death, de‐registration from a practice, last practice data collection, or at the study end date, whichever occurred first. The study flow diagram of participants can be found in the online supplementary *Figure* S1.

Study approval was granted by the Independent Scientific Advisory Committee of the Medicines and Healthcare products Regulatory Agency (protocol 14_ 246) and the MINAP Academic Group.

### Risk factors

Risk factors included in this study were: age, sex, ethnicity, social deprivation, body mass index (BMI), physical activity, smoking, diastolic BP (DBP), systolic BP (SBP), lipid measures (total cholesterol and triglyceride), physiological markers [albumin, creatinine, platelets and white blood cell (WBC) count], comorbidities [diabetes, hypertension, atrial fibrillation (AF) and chronic obstructive pulmonary disease (COPD)] and prescriptions of BP‐lowering and lipid‐regulating drugs.

Baseline risk factors were identified as the closest measurement to index date up to 3 years before and 1 year after index date. All determinants were recorded during consultations in CPRD or HES. Reported ethnicity was used to classify individuals as Caucasian, black, Asian, or other. Social deprivation was measured as quintiles of the index of multiple deprivation, a score calculated based on seven indices of deprivation: income, employment, health and disability, education, barrier to housing and services, crime, and living environment.^14^ Furthermore we classified hypertension as three SBP measurements >140 mmHg and/or use of BP‐lowering medication, obesity as a BMI measurement >30 kg/m^2^, smoking status as never, ex‐ or current smokers, and patient's level of physical activity as recorded in primary care was classified as sedentary lifestyle or active lifestyle. Definitions of all risk factors can be found at https://www.caliberresearch.org/portal/.

### Endpoints

The primary endpoint was incident HF and was based on the first record of HF from CPRD or HES.^4^ Events in CPRD were defined by a diagnosis of HF or diagnosis of chronic left ventricular dysfunction on echocardiogram with READ codes, and in HES by a diagnosis of HF with ICD‐10. Secondary endpoint was the first record of HF, excluding patients with a previous myocardial infarction (MI) event at baseline. READ and ICD‐10 codes for HF and MI definitions can be found in the online supplementary *Table* S1.

### Statistical analysis

Incidence rates of HF (per 1000 person‐years of follow‐up) were estimated by calendar time including 95% confidence intervals (CI), stratified by sex and age category: 55–64, 65–74 and > 75 years.

Missing data in all baseline risk factors were imputed, except comorbidities and prescriptions, using multiple imputation, from the *mice* algorithm in the statistical software package R. We stratified imputations by sex and age category and created 10 imputed datasets. Analyses were performed on the imputed datasets separately and results were pooled using Rubin's rules. Multivariable Cox proportional hazards analysis was used to estimate hazard ratios (HRs) for associations between baseline risk factors and incident HF, separately by sex and age categories for all baseline risk factors. The proportional hazards assumption was verified by assessment of the Schoenfeld residuals. For our secondary analysis, we repeated the above analysis in a subset of individuals without a history of MI. The Bonferroni correction was used to account for multiple testing. We tested for interaction with age categories (55–64, 65–74 and > 75 years) and sex for all associations presented.

We estimated the population attributable risk (PAR) of risk factors for incident HF for: social deprivation, smoking, sedentary lifestyle, obesity and diabetes. To assess the impact of these risk factors, we estimated the PAR (95% CI) with the standard formula: PAR = [P(F)*(HR‐1)]/[1 + P(F)*(HR‐1)] where P(F) is the prevalence of the risk factor in the population and HR the HR of disease due to that risk factor.^15^

In sensitivity analyses, we compared the results after multiple imputation to those based on a complete case analysis and to a subset of individuals not using BP‐lowering medication at baseline. Furthermore, we compared inter‐practice/hospital variation in a frailty Cox proportional hazards model where practice is a random effects variable and we compared associations of risk factors for incident HF stratified by endpoints from different sources of EHR (CPRD and HES).

All analyses were performed using R version 3.2.3.

## Results

### Baseline characteristics

The study cohort included 871 687 individuals aged 55 years or older of whom 47 987 (5.5%) individuals developed incident HF during a median follow‐up of 5.8 years [interquartile range (IQR) 2.7–9.9], with a median time to event of 3.7 years [IQR 1.8–6.4]. A Kaplan–Meier time‐to‐event plot for incident HF can be found in the online supplementary *Figure* S2. Baseline characteristics are presented separately for men (*Table* 1) and women (*Table* 2), stratified by age and incident HF development. Compared to individuals without HF, incident HF patients more often had a higher social deprivation, sedentary lifestyle, higher BMI, higher SBP, and higher creatinine levels, and were more often smokers at baseline. Comorbidities more often occurred in incident HF patients than individuals without HF at baseline; this was similar for both men and women.

**Table 1:** Baseline characteristics stratified by age and heart failure status in men

	55–64 years		65–74 years		>75 years	
	Incident HF patients	Individuals without HF	Incident HF patients	Individuals without HF	Incident HF patients	Individuals without HF
*n*	5408	252 290	8047	80 369	9859	48 672
**Demographics (%)**						
Ethnicity (Caucasian)	96.1	95.1	96.6	95.8	98.2	97.4
Most deprived fifth^a^	26.2	17.2	22.8	18.1	19.4	19.3
**Lifestyle (%)** b						
Smoking						
Current smoking	32.4	27.9	19.7	18.8	12.5	13
Ex‐smoker	31.9	29.3	38.7	36.5	39.5	38.7
Never smoked	35.7	42.8	41.6	44.7	48	48.3
Sedentary lifestyle	43.5	36.4	48.1	41.2	62.3	58.1
**Clinical measures, mean (SD) or median [IQR]** b						
Body mass index (kg/m^2^)	28.5 (5.2)	27.5 (4.5)	27.3 (4.4)	26.6 (4.0)	25.7 (4.0)	25.1 (3.9)
Total cholesterol (mmol/L)	5.4 (1.2)	5.5 (1.0)	5.2 (1.0)	5.3 (1.0)	5.1 (1.0)	5.1 (1.0)
Triglycerides (mmol/L)	1.9 (1.4)	1.8 (1.2)	1.7 (1.1)	1.7 (1.0)	1.5 (0.9)	1.5 (0.9)
SBP (mmHg)	142.0 (19.3)	138.4 (16.9)	146.7 (19.2)	145.4 (18.3)	147.9 (19.8)	146.5 (19.5)
DBP (mmHg)	83.5 (10.2)	83.2 (9.5)	81.4 (9.6)	82.0 (9.4)	79.8 (9.8)	79.6 (9.7)
Heamoglobin (g/dL)	14.7 (1.4)	14.8 (1.2)	14.2 (1.6)	14.3 (1.5)	13.5 (1.7)	13.5 (1.7)
Platelets (10^9/L)	240.7 [80.5]	243.0 [77.4]	229.3 [82.1]	231.1 [80.9]	226.8 [87.1]	230.1 [89.3]
Total WBC count (10^9/L)	7.5 (2.4)	7.0 (2.2)	7.5 (2.6)	7.2 (2.5)	7.6 (2.8)	7.5 (2.7)
Albumin (g/L)	41.8 (3.9)	42.4 (3.6)	40.8 (3.9)	40.9 (3.9)	39.3 (4.3)	39.2 (4.4)
Creatinine (µmol/L)	94.4 [21.6]	93.0 [18.25]	100.0 [25.0]	98.0 [22.35]	104.2 [30.5]	108.3 [35.0]
**Comorbidity (%)** c						
Atrial fibrillation	6.5	0.9	8.8	2.4	11.1	4.6
COPD	22.4	10.2	25.4	13.8	27.7	18.1
Diabetes mellitus	5.5	1.6	5.3	2.3	3.7	2.4
Myocardial infarction	5.3	0.9	4.2	1.3	3.8	1.5
Hypertension	72.1	54.9	80.0	71.6	80.8	72.9
Obesity	42.6	26.6	24.0	17.4	13.7	10.3
**Medication use (%)**						
BP‐lowering medication	37.3	15.9	48.7	26.4	59.0	34.9
Lipid‐regulating drugs	29.6	13.6	26.2	17.2	11.5	10.7

BP, blood pressure; COPD, chronic obstructive pulmonary disease; DBP, diastolic blood pressure; eGFR, estimated glomerular filtration rate; HDL, high‐density lipoprotein; HF, heart failure; IQR, interquartile range; LDL, low‐density lipoprotein; SBP, systolic blood pressure; SD, standard deviation; WBC, white blood cell.

a Assessed by index of multiple deprivation.

b Measurement closest to and within 3 years before baseline.

c Denotes prior medical history of given comorbidity 3 years before baseline, prescription use 3 years before baseline.

**Table 2:** Baseline characteristics stratified by age and heart failure status in women

	55–64 years		65–74 years		>75 years	
	Incident HF patients	Individuals without HF	Incident HF patients	Individuals without HF	Incident HF patients	Individuals without HF
*n*	2878	254 486	6624	94 568	15 171	93 315
**Demographics (%)**						
Ethnicity (Caucasian)	94.8	94.5	96.7	96.0	98.8	98.2
Most deprived fifth^a^	29.4	16.4	25.4	18.6	22.2	19.1
**Lifestyle (%)** b						
Smoking						
Current smoking	26.2	21.3	16.5	14.4	7.3	7.4
Ex‐smoker	23.3	21.1	23.8	22.5	20.7	20.4
Never smoked	50.5	57.6	59.7	63.1	72.0	72.2
Sedentary lifestyle	52.8	41.7	60.3	51.3	75.5	71.3
**Clinical measures, mean (SD) or median [IQR]** b						
Body mass index (kg/m^2^)	29.8 (7.2)	27.3 (5.7)	28.1 (5.8)	26.8 (5.2)	25.5 (5.0)	24.7 (4.7)
Total cholesterol (mmol/L)	5.8 (1.2)	5.8 (1.1)	5.9 (1.2)	6.0 (1.1)	5.8 (1.2)	5.8 (1.2)
Triglycerides (mmol/L)	1.8 (1.1)	1.5 (0.9)	1.8 (1.0)	1.6 (0.9)	1.7 (1.1)	1.6 (0.9)
SBP (mmHg)	144.0 (19.5)	136.3 (17.5)	150.7 (19.8)	147.3 (18.7)	152.0 (20.9)	149.5 (20.8)
DBP (mmHg)	82.8 (9.8)	81.3 (9.2)	82.5 (9.7)	82.4 (9.3)	81.2 (9.9)	80.5 (10.0)
Heamoglobin (g/dL)	13.4 (1.3)	13.5 (1.1)	13.2 (1.4)	13.3 (1.3)	12.7 (1.5)	12.7 (1.5)
Platelets (10^9/L)	268.1 [85.2]	268.7 [87.1]	263.8 [91.2]	263.5 [88.5]	260.6 [96.7]	264.0 [97.4]
Total WBC count (10^9/L)	7.3 (2.3)	6.7 (2.0)	7.3 (2.4)	7.0 (2.3)	7.5 (2.6)	7.3 (2.5)
Albumin (g/L)	41.2 (3.8)	41.9 (3.5)	40.5 (3.8)	40.7 (3.8)	39.0 (4.4)	38.9 (4.5)
Creatinine (µmol/L)	78.9 [18.8]	76.7 [16.5]	84.0 [23.2]	81.0 [20.0]	90.2 [29.3]	86.2 [25.6]
**Comorbidity (%)** c						
Atrial fibrillation	5.4	0.4	7.7	1.4	10.9	3.7
COPD	29.4	13.1	27.4	14.1	24.6	15.2
Diabetes mellitus	6.5	1.1	4.9	1.7	3.4	1.9
Myocardial infarction	2.6	0.2	2.6	0.5	2.3	0.9
Hypertension	82.6	47.6	81.7	72.3	70.2	75.3
Obesity	42.6	26.6	32.4	23.1	16.8	12.7
**Medication use (%)**						
BP‐lowering medication	47.2	18.9	58.6	31.8	69.1	44.2
Lipid‐regulating drugs	22.9	9.4	21.6	14.3	8.8	8.7

BP, blood pressure; COPD, chronic obstructive pulmonary disease; DBP, diastolic blood pressure; eGFR, estimated glomerular filtration rate; HDL, high‐density lipoprotein; HF, heart failure; IQR, interquartile range; LDL, low‐density lipoprotein; SBP, systolic blood pressure; SD, standard deviation; WBC, white blood cell.

a Assessed by index of multiple deprivation.

b Measurement closest to and within 3 years before baseline.

c Denotes prior medical history of given comorbidity 3 years before baseline, prescription use 3 years before baseline.

### Incidence rates

**Figure 1:**
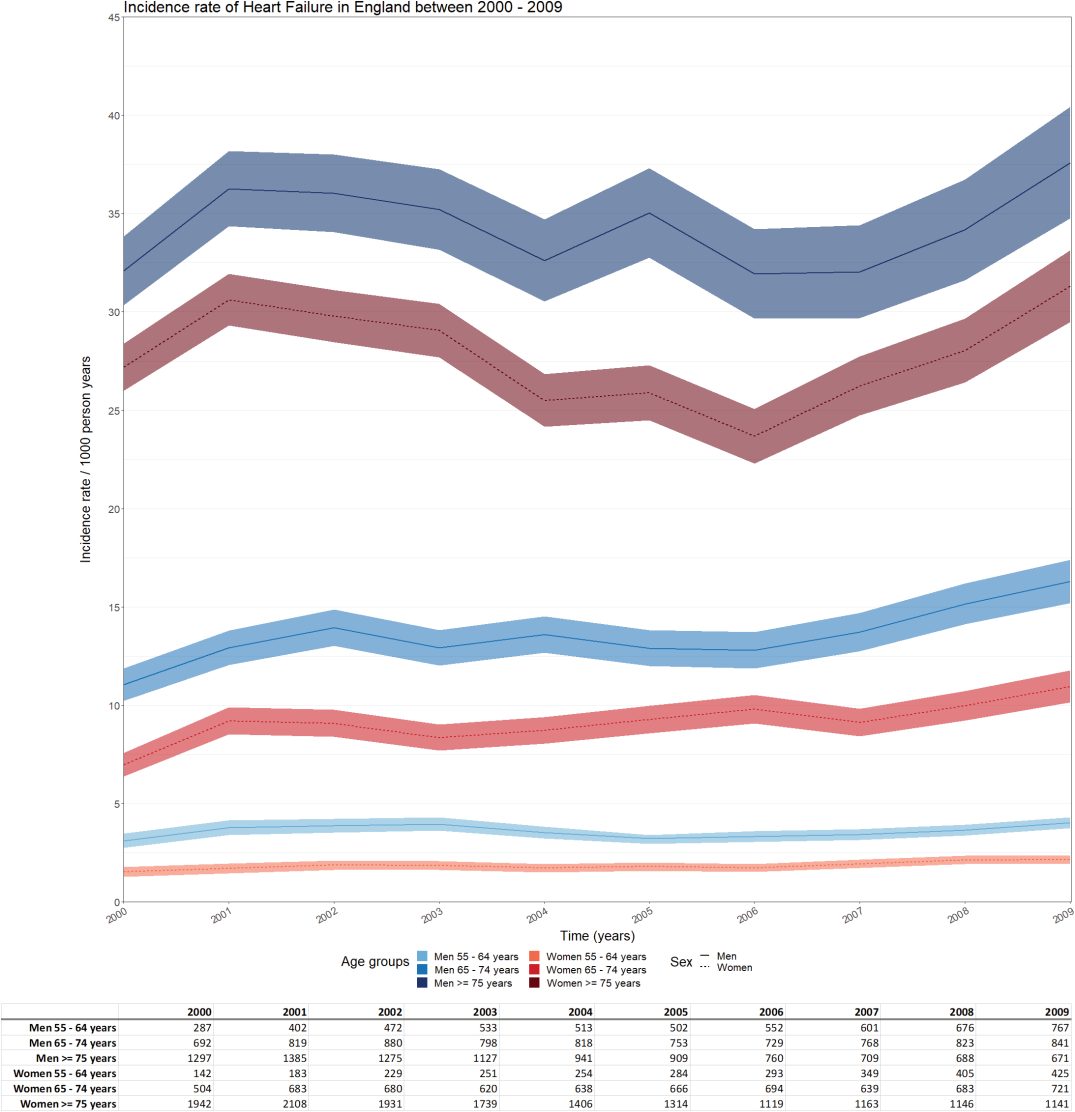
Incidence rate (per 1000 person‐years) of heart failure in England between 2000 and 2009 stratified by age category and sex. Incidence rate/1000 person‐years with 95% confidence interval (band), table with absolute number of cases stratified by age category and sex.

Incidence rates of HF events per 1000 person‐years varied between sexes and age categories. Overall, incidence rates in men were higher than in women (*Figure* 1). Incidence rates were stable over calendar time for men and women aged 55–64 years with a mean incidence rate per 1000 person‐years of 3.6 and 1.9, respectively; these incidence rates increased with older age to an average of 13.6 for men and 9.2 for women at age 65–74 years. The highest incidence rate per 1000 person‐years was observed for the age category >75 years with a mean incidence rate per 1000 person‐years of 34.4 for men and 28.0 for women.

### Risk factors for incident heart failure

**Figure 2:**
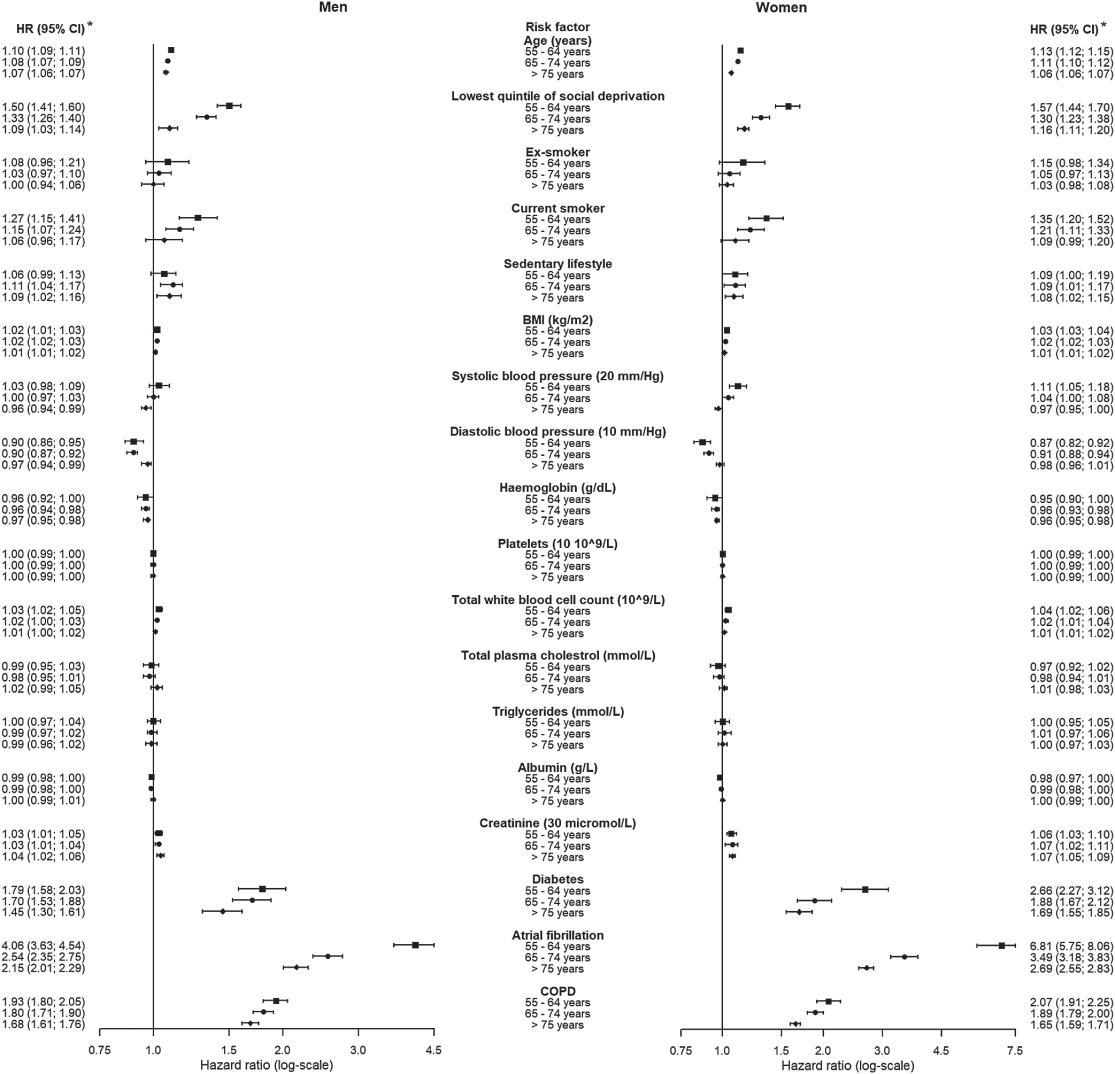
Forest plot of multivariable hazard ratios [95% confidence interval (CI)] of risk factors for incident heart failure, stratified by age and sex. Results of the multivariable model showing independent hazard ratios of other variables shown and further adjusted for ethnicity, blood pressure‐lowering medication and lipid‐regulating drugs and stratified by age and sex. Square boxes = 55–64 years, circle boxes = 65–74 years and diamond boxes = > 75 years. *Hazard ratios were considered statistically significant if *P* < 0.001 (Bonferroni corrected threshold). Patient (events) men: age category 55–64 years *n* (events) = 257 698 (5408), age category 65–74 years *n* (events) = 88 416 (8047), age category >75 years *n* (events) = 58 531 (9859). Patient (events) women: age category 55–64 years *n* (events) = 257 364 (2878), age category 65–74 years *n* (events) = 101 192 (6624), age category >75 years *n* (events) = 108 486 (15171). BMI, body mass index; COPD, chronic obstructive pulmonary disease.

Results from the multivariable Cox proportional hazards models show that diabetes, AF and COPD had the strongest associations with incident HF in men and even stronger associations with HF in women in all age categories, with associations attenuating with older age (*P*‐value for interaction with age < 0.05). In men, we found associations with HF for age, lowest quintile of social deprivation, BMI, haemoglobin, total WBC count and creatinine in all age categories (*Figure* 2). The associations of age, social deprivation, smoking and BP all attenuated in older men (*P*‐value for interaction with age < 0.05), whereas the association of sedentary lifestyle with incident HF was stronger in the older age categories compared to 55–64 year olds (*P*‐value for interaction with age < 0.05).

We found similar associations for women, age, lowest quintile social deprivation, current smoking, sedentary lifestyle, BMI, haemoglobin, total WBC count and creatinine were associated with incident HF in all age categories. However, compared to men, women showed stronger associations of creatinine, diabetes, AF and COPD, these were associated with incident HF in all age categories (*P*‐value for interaction with sex < 0.05) (*Figure* 2). Similar to men, associations of social deprivation, smoking, BP and diabetes attenuated in older women (*P*‐value interaction with age < 0.05).

We found no associations with incident HF in either men or women for platelets, total plasma cholesterol, triglycerides, or albumin (*Figure* 2). We found an association for SBP (per 20 mmHg) for the youngest age category in women (1.11, 95% CI 1.05–1.18), but not for men. Furthermore, SBP was inversely associated in the oldest age category for both sexes (0.96, 95% CI 0.94–0.99 and 0.97, 95% CI 0.96–1.00, respectively). DBP (per 10 mmHg) was inversely associated with incident HF in the two younger age categories, whereas no association in the oldest age group was observed (*Figure* 2). Overall results from the multivariable Cox proportional hazards model, men and women and all ages combined, are shown in the online supplementary *Figure S3*. When patients with and without a history of MI were analysed, similar HRs were found for the associations between risk factors and incident HF in men and women (online supplementary *Figures* S4 and S5), with a trend towards a positive association of total cholesterol with HF, though not significant. When we added history of MI to the main model, it did not change the observed associations of other risk factors (data not shown). When we compared individuals using BP‐lowering medication with those who were not, we observed an attenuation of most associations in individuals not prescribed BP‐lowering medication, except for SBP and diabetes, the associations of these risk factors with incident HF became stronger in all age categories for both men and women (online supplementary *Figures* S6 and S7).

### Relative contribution of modifiable risk factors and comorbidities

The largest proportion of male HF cases that could be prevented was if COPD, AF and hypertension would not occur in the population (*Table* 3). A smaller proportion of cases could be prevented by the modifiable lifestyle factors obesity, diabetes and current smoking.

**Table 3:** Relative contributions of risk factors for incident heart failure stratified by age in men

Risk factors	Hazard ratio (95% CI)^a^	Prevalence	Relative contribution (95% CI)
55–64 years			
COPD	1.93 (1.81–2.06)	0.22	17.24 (15.36–19.19)
Atrial fibrillation	4.04 (3.62–4.52)	0.07	16.50 (14.55–18.62)
Obesity^b^	1.21 (1.11–1.31)	0.48	9.07 (4.97–12.84)
Sedentary lifestyle	1.06 (0.99–1.14)	0.44	N.E.
Diabetes	1.85 (1.64–2.10)	0.06	4.47 (3.40–5.70)
Current smokers	1.27 (1.14–1.40)	0.32	8.04 (4.34–11.47)
Hypertension	1.14 (1.07–1.22)	0.72	9.17 (4.80–13.69)
65–74 years			
COPD	1.81 (1.72–1.90)	0.25	17.06 (15.46–18.61)
Atrial fibrillation	2.54 (2.35–2.75)	0.09	11.93 (10.62–13.34)
Obesity^b^	1.25 (1.18–1.34)	0.24	5.66 (4.14–7.54)
Sedentary lifestyle	1.11 (1.05–1.18)	0.48	5.03 (2.35–7.97)
Diabetes	1.73 (1.57–1.92)	0.05	3.72 (2.93–4.65)
Current smokers	1.15 (1.07–1.24)	0.20	2.87 (1.36–4.51)
Hypertension	1.03 (0.97–1.09)	0.80	N.E.
>75 years			
COPD	1.69 (1.61–1.76)	0.28	16.05 (14.45–17.39)
Atrial fibrillation	2.16 (2.02–2.30)	0.11	11.41 (10.17–12.61)
Obesity^b^	1.15 (1.07–1.25)	0.14	2.01 (0.95–3.31)
Sedentary lifestyle	1.09 (1.03–1.16)	0.62	5.31 (1.83–9.06)
Diabetes	1.45 (1.31–1.62)	0.04	1.64 (1.13–2.24)
Current smokers	1.05 (0.95–1.16)	0.19	N.E.
Hypertension	1.10 (1.05–1.15)	0.81	7.48 (3.88–10.81)

CI, confidence interval; COPD, chronic obstructive pulmonary disease; N.E., not estimable.

Hazard ratios were considered statistically significant if *P* <0.001 (Bonferroni corrected threshold).

a Independent hazard ratios of other variables shown and further adjusted for age, haemoglobin, platelets, total white blood cell count, total cholesterol, triglycerides, albumin, creatinine, ethnicity, smoking habits, index of multiple deprivation, blood pressure‐lowering medication and lipid‐lowering drugs.

b Obesity, body mass index ≥ 30 kg/m^2^.

Relative contributions of risk factors to incident HF appeared to be stronger in women compared to men. In women, the largest proportion of HF cases that could be prevented by modifiable risk factors were COPD and AF, but not hypertension. Similar to men, obesity and diabetes could prevent a smaller proportion of HF cases (*Table* 4). In both men and women the relative contributions attenuated with older age, whereas the relative contribution of sedentary lifestyle remained similar across age categories.

**Table 4:** Relative contributions of risk factors for incident heart failure stratified by age in women

Risk factors	Hazard ratio (95% CI)^a^	Prevalence	Relative contribution (95% CI)
55–64 years			
COPD	2.07 (1.91–2.25)	0.29	23.93 (21.11–26.87)
Atrial fibrillation	6.78 (5.73–8.01)	0.05	23.79 (20.35–27.46)
Obesity^b^	1.39 (1.25–1.54)	0.43	14.25 (9.62–18.70)
Sedentary lifestyle	1.12 (1.03–1.22)	0.52	5.96 (1.56–10.41)
Diabetes	2.77 (2.36–3.24)	0.07	10.32 (8.12–12.71)
Current smokers	1.33 (1.18–1.49)	0.26	7.96 (4.50–11.38)
Hypertension	1.09 (1.00–1.19)	0.83	N.E.
65–74 years			
COPD	1.89 (1.79–2.00)	0.27	19.61 (17.79–21.51)
Atrial fibrillation	3.49 (3.18–3.83)	0.08	16.09 (14.37–17.89)
Obesity^b^	1.25 (1.17–1.34)	0.32	7.49 (5.22–9.92)
Sedentary lifestyle	1.10 (1.02–1.18)	0.60	5.69 (1.19–9.79)
Diabetes	1.91 (1.70–2.15)	0.05	4.27 (3.32–5.02)
Current smokers	1.21 (1.11–1.32)	0.24	3.35 (1.78–5.02)
Hypertension	0.98 (0.92–1.04)	0.82	N.E.
>75 years			
COPD	1.65 (1.59–1.71)	0.25	13.79 (12.67–14.87)
Atrial fibrillation	2.69 (2.55–2.84)	0.11	15.56 (14.45–16.71)
Obesity^b^	1.14 (1.08–1.20)	0.17	2.30 (1.33–3.25)
Sedentary lifestyle	1.09 (1.02–1.16)	0.76	6.36 (1.49–10.78)
Diabetes	1.70 (1.56–1.86)	0.03	2.32 (1.87–2.84)
Current smokers	1.08 (0.99–1.19)	0.07	N.E.
Hypertension	1.02 (0.99–1.07)	0.70	N.E.

CI, confidence interval; COPD, chronic obstructive pulmonary disease; N.E., not estimable.

Hazard ratios were considered statistically significant if *P* <0.001 (Bonferroni corrected threshold).

a Independent hazard ratios of other variables shown and further adjusted for age, haemoglobin, platelets, total white blood cell count, total cholesterol, triglycerides, albumin, creatinine, ethnicity, smoking habits, index of multiple deprivation, blood pressure‐lowering medication and lipid‐lowering drugs.

b Obesity: body mass index ≥ 30 kg/m^2^.

### Sensitivity analysis

Patient characteristics were similar between imputed data and complete case data for men and women (online supplementary *Tables* S2 and S3). Sensitivity analysis showed that a complete case analysis yielded similar directions of associations for risk factors with incident HF in both men and women (online supplementary *Tables* S4 and S5); however, associations were attenuated in the imputed data analysis. General practice variability had no effect on the overall associations in men and women (online supplementary *Tables* S6 and S7), since the random effects models resulted in near identical estimates to our main analysis. Lastly, analyses stratified by different sources of EHR showed that the associations of social deprivation, current smoking and diabetes with incident HF were stronger in HES cases compared to CPRD, whereas the association of AF was stronger in younger (55–65 years) men and women in CPRD compared to HES (online supplementary *Tables* S8 and S9). Overall, the analyses were comparable with our main analysis.

## Discussion

In this large population‐based cohort study using linked EHRs, we investigated the association of risk factors with the development of HF. We found independent associations of diabetes, AF, COPD, age, social deprivation, modifiable lifestyle factors and inflammatory markers, but not SBP, with incident HF, in a population using BP‐lowering and lipid‐regulating medication.

In England, we found higher incidence rates for men and the elderly (>75 years) which were stable in the period of 2000–2005, though increasing from 2006 onwards for all categories. Previous studies have reported sex‐ and/or age‐specific incidence rates of HF and indicate that the incidence of HF is stable over time, whereas others suggest it might be increasing or even decreasing.^16^, ^17^, ^18^, ^19^, ^20^, ^21^ These differences might be reflected in a varying follow‐up time, diverse patient populations, diversity in quality of data, lack of distinction of incidence rates based on both age and gender, and regional or cultural differences underlying these incidence rates.

### Risk factors for incident heart failure

We confirmed several associations of risk factors with HF, such as diabetes, BMI, and smoking. Our study supports and contributes to previous studies in CALIBER,^21^, ^22^, ^23^, ^24^, ^25^ which have shown associations of these risk factors with a range of CVDs. We observed similar patterns of association between men and women as well as attenuation of the associations of risk factors with HF at older age. Compared to men, women showed stronger associations of modifiable lifestyle factors, such as smoking, a sedentary lifestyle and diabetes, with incident HF. This could reflect a different aetiology between men and women in the development of HF.

We found no, or weak, independent association between SBP and incident HF in our multivariable analyses. This contrasts with papers reporting on the association of SBP with incident HF.^5^, ^6^ However, similar associations between SBP and incident HF, as previously reported,^6^ could be reproduced by excluding individuals using BP‐lowering medication in our analyses. This reinforces the importance of treating high BP accordingly.

Our results show that in a population with high prescription rates of BP‐lowering medication, smaller independent associations of other risk factors become more evident. For example, we found levels of total WBC count independently associated with HF, this could indicate an underlying inflammatory process leading to HF.^26^, ^27^ Inflammation could be triggered by comorbidities such as diabetes or obesity or via endothelial dysfunction and atherosclerosis from an underlying heart disease; however, it remains to be investigated how inflammation and HF interact exactly. Similar results have recently been reported for other CVDs.^24^ Additionally, we found an association of creatinine and an inverse association of haemoglobin with incident HF. Low haemoglobin, or anaemia, and raised creatinine levels are frequently observed among HF patients and are associated with worse outcomes and increased mortality.^28^, ^29^ Lastly, our results show an inverse association of DBP with incident HF. This is likely due to reversed causality induced by the relatively old age of our study population [median age 61.5 years (IQR 55–71.9)]; it is known that DBP is lower in the elderly and is associated with worse survival.^30^, ^31^

Observing the substantial prevalence of modifiable risk factors and comorbidities, such as COPD, AF, obesity, a sedentary lifestyle and smoking, our results suggest that preventive strategies could be an opportunity to reduce the risk of incident HF. Previous research has already shown that adherence to a healthy lifestyle reduces the lifetime risk of HF.^32^, ^33^ Future studies should verify these results in population‐based studies and focus should be directed to implicating effective preventive strategies in clinical practice.

### Study strengths and limitations

Strengths of this study are the linkage of multiple EHR sources, which allowed for the collection of a large representative sample of 871 687 individuals across England and studying a large population of HF patients. Previous studies have shown the feasibility and validity of routinely collected data in CPRD and HES.^34^, ^35^ However, several limitations of this study should be considered when interpreting these findings. First, due to the nature of EHR, the accuracy and amount of detailed information recorded are limited, though findings based on the multiple imputed dataset showed a similar direction of association compared to complete case analysis. Residual confounding may still exist. Second, we were unable to differentiate between HF phenotypes, since there was no access to detailed echocardiography estimates to assess systolic function. This is likely to conceal a greater degree of heterogeneity. Third, all measurements are prone to measurement error and/or misclassification. To define HF, we used data from two different EHR sources, each having their own measurement error. Yet, associations were similar between CPRD and HES cases in our sensitivity analysis, and others have delivered evidence of the validity of using linked EHRs.^4^, ^36^

## Conclusions

In this large population‐based cohort study using linked EHRs in England, we observed that diabetes, AF, COPD, age, social deprivation, modifiable lifestyle factors such as smoking, sedentary lifestyle, BMI, and physiological measures such as haemoglobin, total WBC count and creatinine were associated with incident HF across age‐ and sex‐specific groups. Mainly modifiable risk factors and comorbidities are of interest, considering a substantial PAR. This highlights the importance of preventive strategies targeting modifiable lifestyle risk factors for HF, besides BP management.

## Supporting information


**Figure S1.** Flowchart of the study population.
**Figure S2.** Kaplan–Meier time‐to‐event for incident heart failure.
**Figure S3.** Risk factors associated with incident heart failure.
**Figure S4.** Risk factors associated with incident heart failure in men stratified by age and prior myocardial infarction.
**Figure S5.** Risk factors associated with incident heart failure in women stratified by age and prior myocardial infarction.
**Figure S6.** Risk factors associated with incident heart failure in men stratified by age and blood pressure lowering medication.
**Figure S7.** Risk factors associated with incident heart failure in women stratified by age and blood pressure lowering medication.
**Table S1.** Overview of READ and ICD‐10 codes used to identify heart failure and myocardial infarction in CPRD and HES data sources.
**Table S2.** Complete case baseline characteristics stratified by age in men.
**Table S3.** Complete case baseline characteristics stratified by age in women.
**Table S4.** Complete case analysis for risk factors associated with incident heart failure stratified by age in men.
**Table S5.** Complete case analysis for risk factors associated with incident heart failure stratified by age in women.
**Table S6.** Evaluation of heterogeneity at practice level for the association of risk factors with heart failure stratified by age in men.
**Table S7.** Evaluation of heterogeneity at practice level for the association of risk factors with heart failure stratified by age in women.
**Table S8.** Associations of risk factors with incident heart failure stratified by age and endpoints from different sources of EHR in men.
**Table S9.** Associations of risk factors with incident heart failure stratified by age and endpoints from different sources of EHR in women.
