# Supplementary material for: Risk factors for incident heart failure in age‐ and sex‐specific strata: a population‐based cohort using linked electronic health records
Source: Eur J Heart Fail. 2019 Jan 7;21(10):1197–206. doi: 10.1002/ejhf.1350 (PMC7074015; doi:10.1002/ejhf.1350)
Supplement: Supplementary file 1 — Figure S1. Flowchart of the study population. Figure S2. Kaplan–Meier time‐to‐event for incident heart failure. Figure S3. Risk factors associated with incident heart failure. Figure S4. Risk factors associated with incident heart failure in men stratified by age and prior myocardial infarction. Figure S5. Risk factors associated with incident heart failure in women stratified by age and prior myocardial infarction. Figure S6. Risk factors associated with incident heart failure in men stratified by age and blood pressure lowering medication. Figure S7. Risk factors associated with incident heart failure in women stratified by age and blood pressure lowering medication. Table S1. Overview of READ and ICD‐10 codes used to identify heart failure and myocardial infarction in CPRD and HES data sources. Table S2. Complete case baseline characteristics stratified by age in men. Table S3. Complete case baseline characteristics stratified by age in women. Table S4. Complete case analysis for risk factors associated with incident heart failure stratified by age in men. Table S5. Complete case analysis for risk factors associated with incident heart failure stratified by age in women. Table S6. Evaluation of heterogeneity at practice level for the association of risk factors with heart failure stratified by age in men. Table S7. Evaluation of heterogeneity at practice level for the association of risk factors with heart failure stratified by age in women. Table S8. Associations of risk factors with incident heart failure stratified by age and endpoints from different sources of EHR in men. Table S9. Associations of risk factors with incident heart failure stratified by age and endpoints from different sources of EHR in women. [file EJHF-21-1197-s001.docx]

**Risk Factors for Incident Heart Failure in Age and Sex Specific Strata: a Population-Based cohort using linked Electronic Health Records**

Alicia Uijl^1,2^, Stefan Koudstaal^2,3^, Kenan Direk^2^, Spiros Denaxas^2^, Rolf H.H. Groenwold^1, 2^, Amivita Banerjee^2^, Arno W. Hoes^1,2^, Harry Hemingway^2,4^, Folkert W. Asselbergs^2, 3, 5^

1. Julius Center for Health Sciences and Primary Care, University Medical Center Utrecht, Utrecht University, the Netherlands

2. Farr Institute of Health Informatics Research, Institute of Health Informatics, University College London, United Kingdom

3. Department of Cardiology, Division Heart & Lungs, University Medical Center Utrecht, Utrecht University, the Netherlands

4. The National Institute for Health Research, Biomedical Research Centre, University College London Hospitals NHS Foundation Trust

5. Institute of Cardiovascular Science, Faculty of Population Health Sciences, University College London, United Kingdom

**Supplementary material**

**Supplementary Figure 1 – Flowchart of the study population**

Legend: CALIBER = Cardiovascular disease research using Linked Bespoke studies and Electronic health Records

**Supplementary Figure 2 – Kaplan–Meier time-to-event for incident heart failure**

Legend: Kaplan–Meier time-to-event curve stratified for age: 55 - 65 year, 65 - 75 years and > 75 years.

**Supplementary Figure 3 - Risk factors associated with incident heart failure**

Legend: Independent HRs for all individuals, further adjusted for ethnicity, blood pressure lowering medication and lipid regulating drugs. Lowest quintile of social deprivation assessed by index of multiple deprivation, HR (95% CI) = Hazard Ratio (95% Confidence Interval), SBP = Systolic Blood Pressure, DBP = Diastolic Blood Pressure, total WBC count = total White Blood Cell count. Hazard ratios were considered statistically significant if p-value < 0.001 (Bonferroni corrected threshold).

**Supplementary Figure 4 - Risk factors associated with incident heart failure in men stratified by age and prior myocardial infarction**

Legend: Independent HRs in a subset of individuals with and without prior MI, further adjusted for ethnicity, blood pressure lowering medication and lipid regulating drugs. Lowest quintile of social deprivation assessed by index of multiple deprivation, MI = Myocardial Infarction, HR (95% CI) = Hazard Ratio (95% Confidence Interval), SBP = Systolic Blood Pressure, DBP = Diastolic Blood Pressure, total WBC count = total White Blood Cell count. Patient events with prior MI: Age category 55-64 years n (events) = 2,426 (285) , age category 65-74 years n (events) = 1,362 (340), age category >75 years n (events) = 1,090 (372). Patient events without prior MI: Age category 55-64 years n (events) = 255,272 (5,123), age category 65-74 years n (events) = 87,054 (7,707), age category >75 years n (events) = 57,441 (9,487). Hazard ratios were considered statistically significant if p-value < 0.001 (Bonferroni corrected threshold).

**Supplementary Figure 5 - Risk factors associated with incident heart failure in women stratified by age and prior myocardial infarction**

Legend: Independent HRs in subset of individuals with and without prior MI, further adjusted for ethnicity, blood pressure lowering medication and lipid regulating drugs. Lowest quintile of social deprivation assessed by index of multiple deprivation, MI = Myocardial Infarction, HR (95% CI) = Hazard Ratio (95% Confidence Interval), SBP = Systolic Blood Pressure, DBP = Diastolic Blood Pressure, total WBC count = total White Blood Cell count. Patient events with prior MI: Age category 55-64 years n (events) = 638 (76), age category 65-74 years n (events) = 688 (175), age category >75 years n (events) = 1,153 (349). Patient events without prior MI: Age category 55-64 years n (events) = 256,726 (2,808), age category 65-74 years n (events) = 100,504 (6,449), age category >75 years n (events) = 107,333 (14,822). Hazard ratios were considered statistically significant if p-value < 0.001 (Bonferroni corrected threshold).

**Supplementary Figure 6 - Risk factors associated with incident heart failure in men stratified by age and blood pressure lowering medication**

Legend: Independent HRs in subset of individuals with and without blood pressure lowering medication, further adjusted for ethnicity and lipid regulating drugs. Lowest quintile of social deprivation assessed by index of multiple deprivation, HR (95% CI) = Hazard Ratio (95% Confidence Interval), SBP = Systolic Blood Pressure, DBP = Diastolic Blood Pressure, total WBC count = total White Blood Cell count. Patient events with blood pressure lowering medication: Age category 55-64 years n (events) = 68,524 (2,921), age category 65-74 years n (events) = 36,768 (5,090), age category >75 years n (events) = 28,700 (6,725). Patient events without blood pressure lowering medication: Age category 55-64 years n (events) = 189,174 (2,487), age category 65-74 years n (events) = 51,648 (2,957), age category >75 years n (events) = 29,831 (3,134). Hazard ratios were considered statistically significant if p-value < 0.001 (Bonferroni corrected threshold).

**Supplementary Figure 7 - Risk factors associated with incident heart failure in women stratified by age and blood pressure lowering medication**

Legend: Independent HRs in subset of individuals with and without blood pressure lowering medication, further adjusted for ethnicity and lipid regulating drugs. Lowest quintile of social deprivation assessed by index of multiple deprivation, HR (95% CI) = Hazard Ratio (95% Confidence Interval), SBP = Systolic Blood Pressure, DBP = Diastolic Blood Pressure, total WBC count = total White Blood Cell count. Patient events with blood pressure lowering medication: Age category 55-64 years n (events) = 69,912 (1659), age category 65-74 years n (events) = 43,712 (4490), age category >75 years n (events) = 60,311 (11,428). Patient events without blood pressure lowering medication: Age category 55-64 years n (events) = 187,452 (1,219), age category 65-74 years n (events) = 57,480 (2,134), age category >75 years n (events) = 48,175 (3,743). Hazard ratios were considered statistically significant if p-value < 0.001 (Bonferroni corrected threshold).

**Supplementary Figure 1**

Individuals with follow-up in the period 2000 - 2010

(n = 2,044,597)

Individuals in CALIBER

(n = 2,134,615)

Individuals included

(n = 871,687)

Individuals without follow-up in the period 2000 - 2010

(n = 90,018)

Individuals excluded:

- < 55 years at start follow-up

(n = 1,138,413)

- < 1 year of follow-up prior to study entry (n = 10,372)
- History of heart failure

(n = 23,482)

- Other (n = 643)

**Supplementary Figure 2**

**
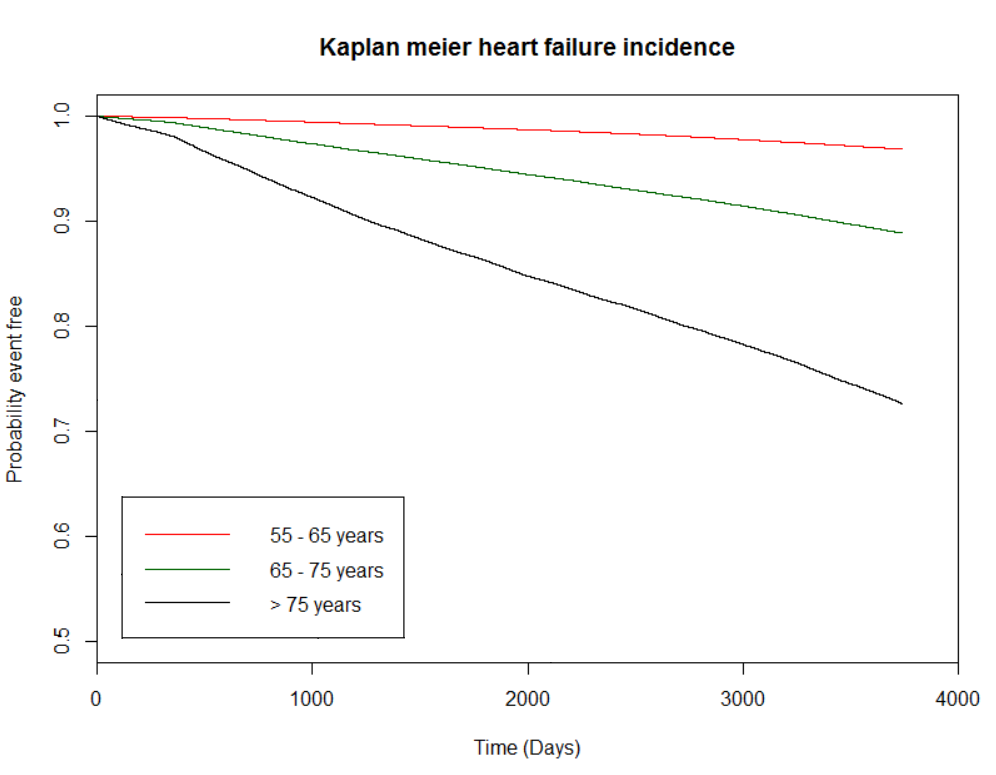
**

**Supplementary Figure 3**

**
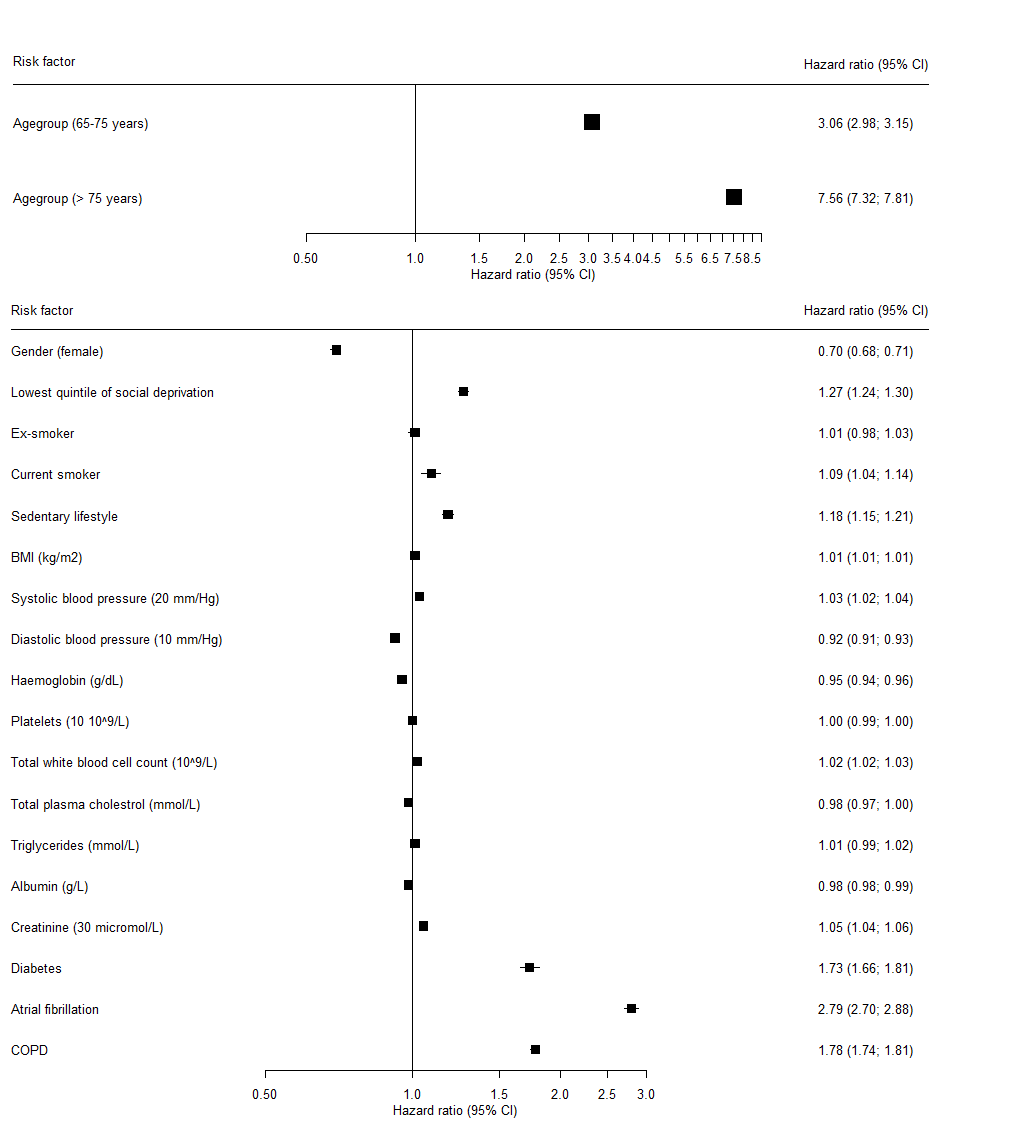
**

**
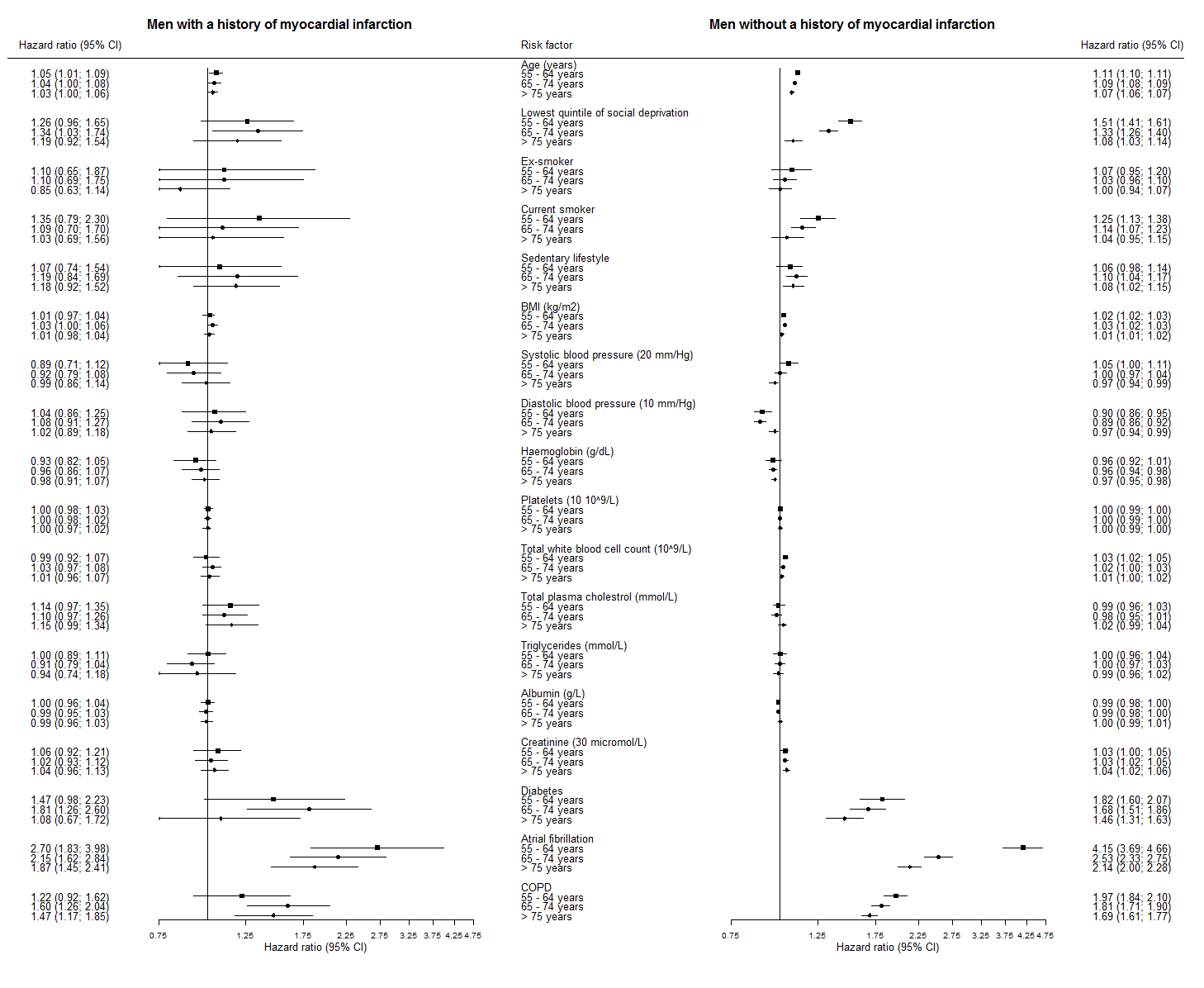
Supplementary Figure 4**

**
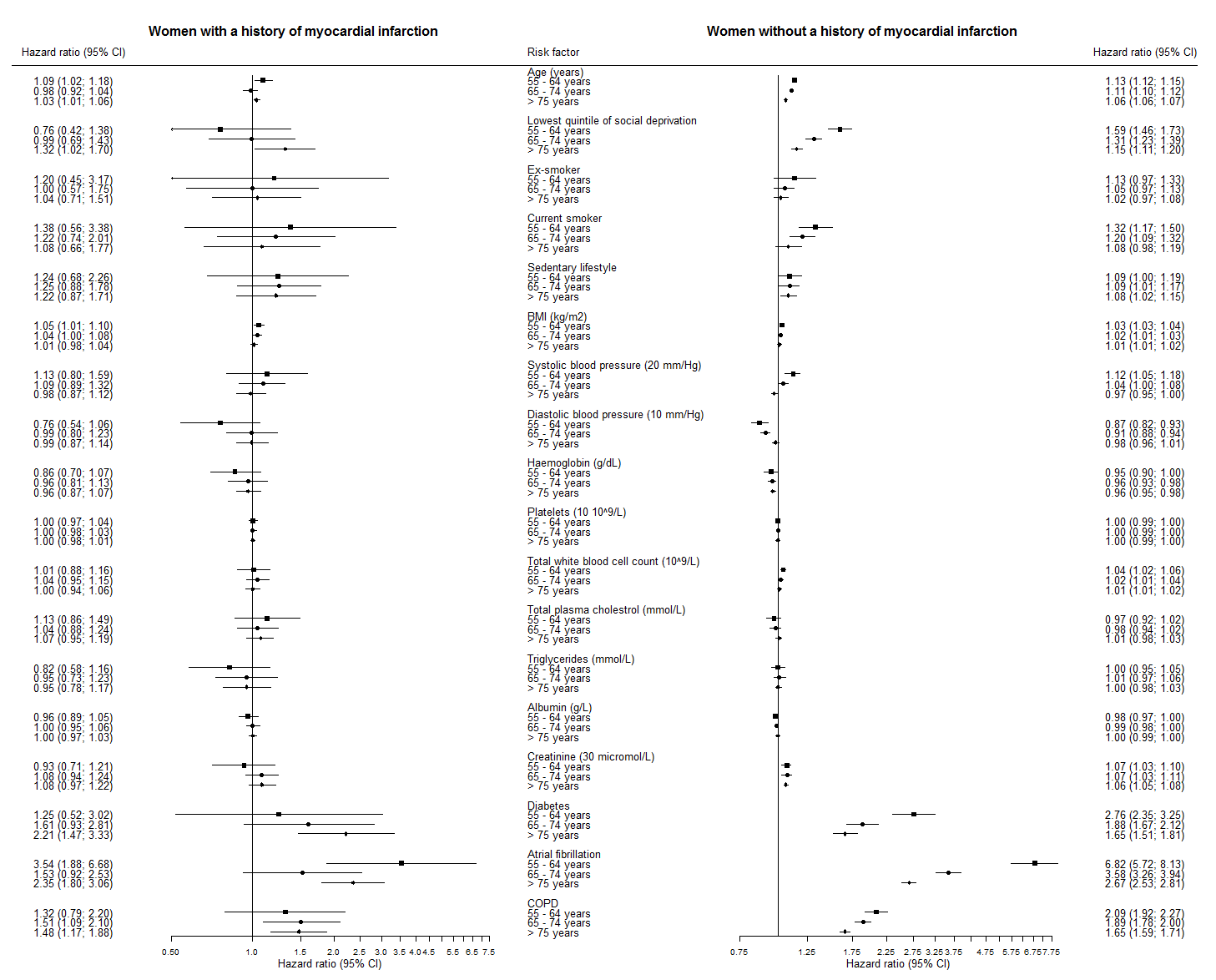
Supplementary Figure 5**

**
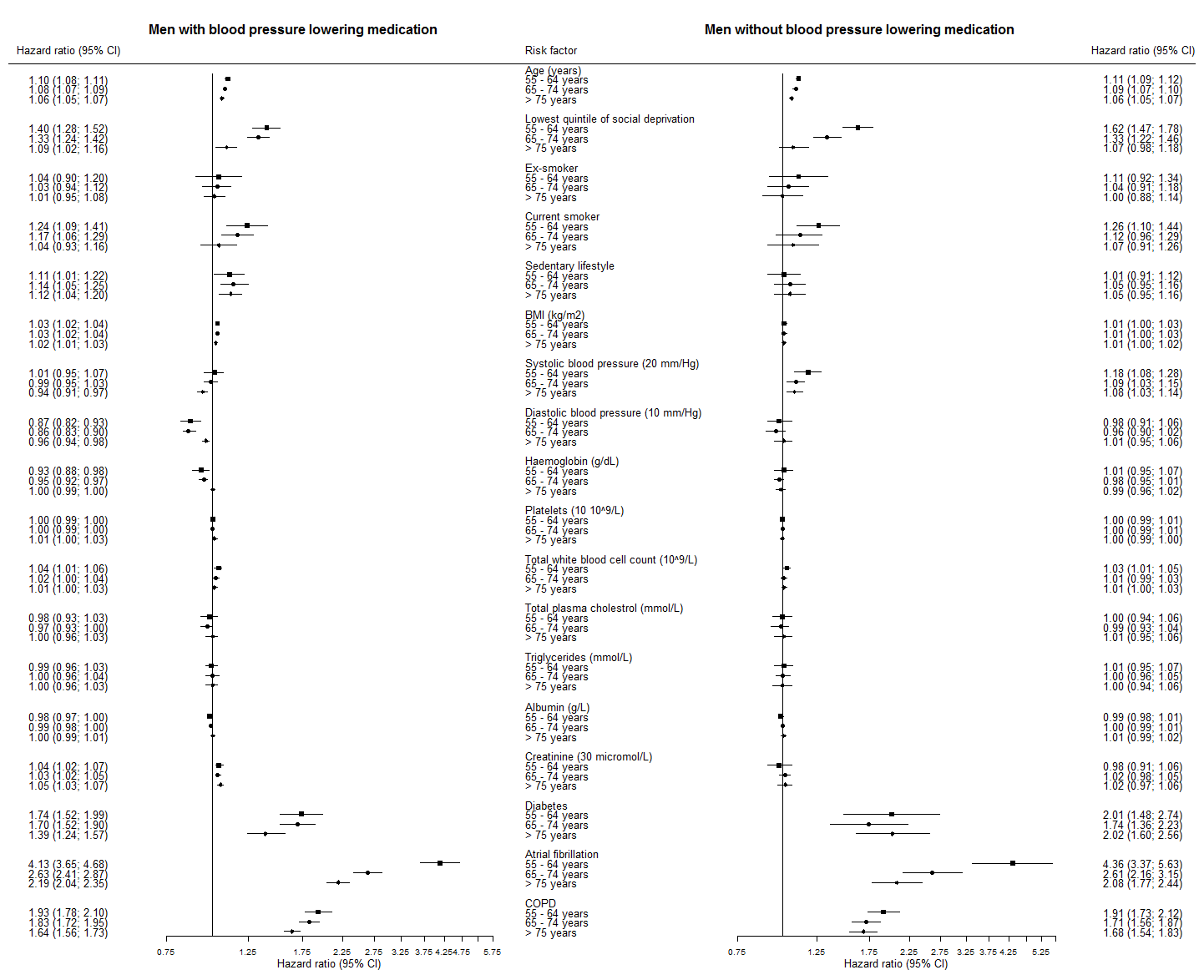
Supplementary Figure 6**

**
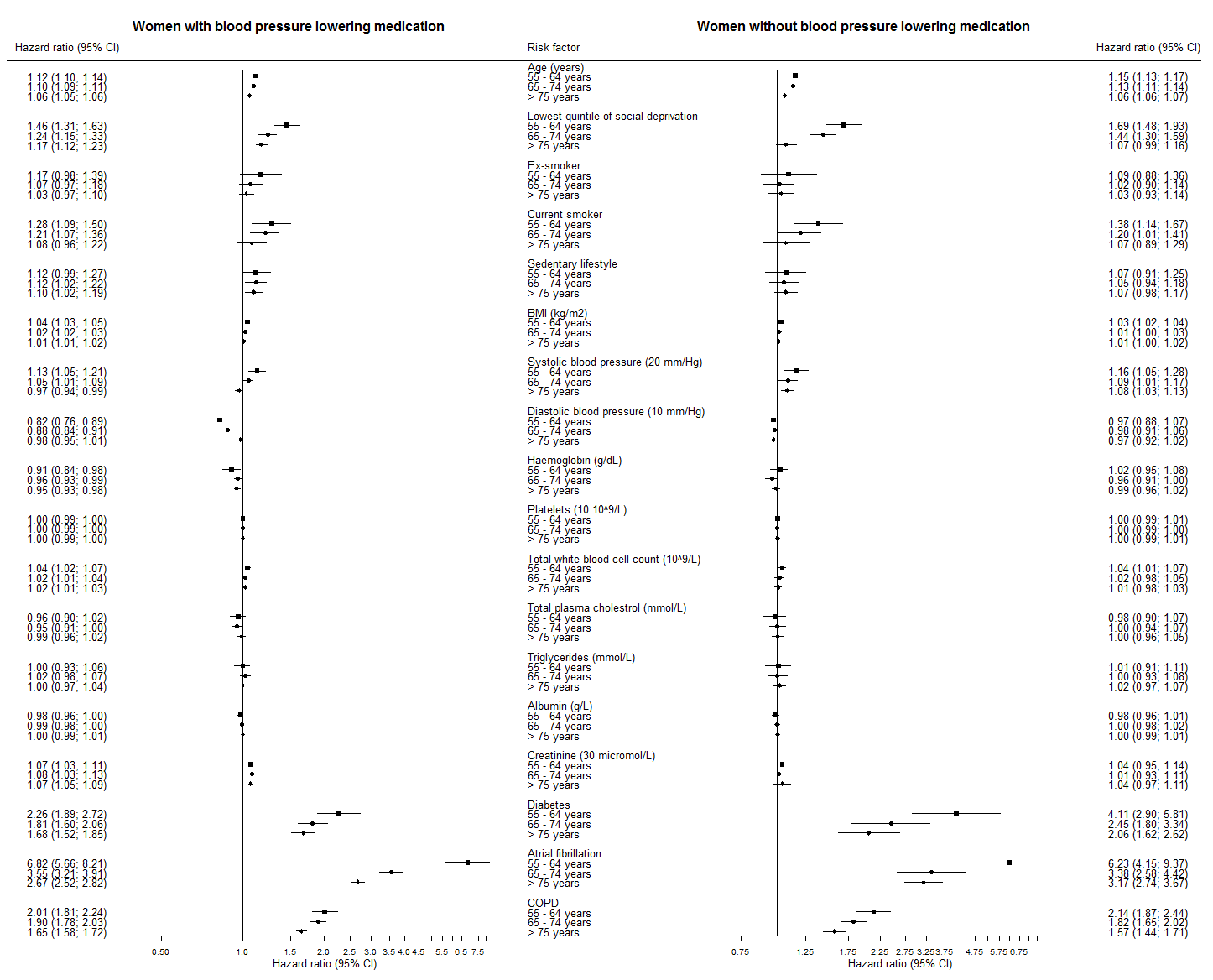
Supplementary Figure 7**

**Supplementary Table 1 - Overview of READ and ICD-10 codes used to identify heart failure and myocardial infarction in CPRD and HES data sources**

|  | CPRD  READ codes | HES  ICD 10 |
| --- | --- | --- |
| Heart failure | G580400, G210.00, G210000, G210100, G211100, G21z100, G230.00, G232.00, G234.00, G1yz100, 1O1..00, 662W.00, 662p.00, 8B29.00, 8H2S.00, 9Or0.00, G400.00, G41z.11, G554000**,** G554011, G58..00, G58..11, G580.00, G580.11, G580.12, G580.13, G580.14, G580000, G580100, G580200, G580300, G581.00, G581.11, G581.13, G581000, G582.00, G58z.00, G58z.12, G5yy900, G5yyA00, R2y1000 | I110, I130, I132,  I260, I50 |
| Non-fatal acute myocardial infarction | G30X000, G307100, 323..00, 3233.00, 3234.00, 3235.00, 3236.00, 323Z.00, 889A.00, G30..00, G30..12, G30..13, G30..15, G30..16, G300.00, G301.00, G301000, G301100, G301z00, G302.00, G303.00, G304.00, G305.00, G306.00, G307.00, G307000, G308.00, G309.00, G30B.00, G30X.00, G30y.00, G30y000, G30y100, G30y200, G30yz00, G30z.00, G31y100, G38..00, G380.00, G381.00, G384.00, G38z.00, Gyu3400 | I21 |

Details of how these codes are defined can be found online at http://www.caliberresearch.org/portal/. CPRD = Clinical Practice Research Datalink; HES = Hospital Episode Statistics.

**Supplementary Table 2 – Complete case baseline characteristics stratified by age in men**

|  | **55 – 64 years** | | **65 – 74 years** | | **> 75 years** | | | **% missing HF subjects** | **% missing**  **non HF subjects** |
| --- | --- | --- | --- | --- | --- | --- | --- | --- | --- |
|  | HF subjects | Non HF subjects | HF subjects | Non HF subjects | HF subjects | Non HF subjects | |  |  |
| Number of patients | 5408 | 252290 | 8047 | 80369 | 9859 | 48672 | | 23314 | 381331 |
| **Demographics** |  |  |  |  |  |  | |  |  |
| Ethnicity (% Caucasian) | 96.3 | 95.1 | 96.8 | 95.8 | 98.3 | 97.4 | | 18.3 | 42.0 |
| Most deprived fifth (%)* | 26.1 | 17.1 | 22.8 | 18.0 | 19.4 | 19.2 | | 0.24 | 0.35 |
| **Lifestyle (%) †** |  |  |  |  |  |  | |  |  |
| Smoking |  |  |  |  |  |  | | 75.3 | 62.9 |
| Current Smoking | 41.7 | 27.9 | 23.0 | 18.0 | 12.3 | 12.2 | | n/a | n/a |
| Ex-smoker | 31.6 | 29.9 | 40.4 | 38.1 | 40 | 40.7 | | n/a | n/a |
| Never smoked | 26.7 | 42.3 | 36.6 | 43.9 | 47.7 | 47.2 | | n/a | n/a |
| Sedentary lifestyle | 51.1 | 37.1 | 55.2 | 40.9 | 64.1 | 57.1 | | 79.2 | 80.3 |
| **Clinical measures in mean (sd)**  **or median [IQR] †** | | |  |  |  |  | |  |  |
| Body Mass Index (kg/m^2^) | 29.5 (5.7) | 27.9 (4.7) | 28.0 (4.6) | 26.8 (4.1) | 26.2 (4.1) | 25.4 (3.9) | | 55.7 | 58.6 |
| Total cholesterol (mmol/L) | 5.3 (1.4) | 5.4 (1.1) | 5.1 (1.0) | 5.2 (1.0) | 5.0 (1.1) | 4.9 (1.1) | | 66.8 | 68.4 |
| Triglycerides (mmol /L) | 2.1 (1.4) | 1.9 (1.3) | 1.8 (1.1) | 1.7 (1.0) | 1.6 (1.0) | 1.5 (0.9) | | 78.9 | 76.6 |
| LDL cholesterol (mmol/L) | 3.3 (1.0) | 3.3 (1.0) | 3.2 (1.0) | 3.2 (1.0) | 3.1 (1.1) | 2.9 (1.0) | | 85.2 | 80.6 |
| HDL cholesterol (mmol/L) | 1.2 (0.4) | 1.3 (0.4) | 1.3 (0.6) | 1.3 (0.4) | 1.3 (0.5) | 1.3 (0.4) | | 84.1 | 79.3 |
| SBP (mmHg) | 142.9 (19.9) | 139.1 (17.2) | 147.1 (19.4) | 146.0 (18.4) | 148.3 (19.9) | 147.0 (19.5) | | 23.2 | 34.1 |
| DBP (mmHg) | 83.7 (10.5) | 83.6 (9.6) | 81.4 (9.6) | 82.1 (9.5) | 79.8 (9.8) | 79.7 (9.7) | | 23.2 | 34.1 |
| Heamoglobin (g/dL) | 14.5 (1.6) | 14.8 (1.2) | 14.0 (1.7) | 14.3 (1.5) | 13.4 (1.7) | 13.5 (1.7) | | 68.5 | 71.2 |
| Platelets (10^9/L) | 239.0 [87.0] | 243.0 [76.5] | 224.5 [83.0] | 230.0 [80.0] | 223.0 [ 85.3] | | 230.0 [88.0] | 73.2 | 73.8 |
| Albumin (g/L) | 41.2 (4.0) | 42.6 (3.6) | 40.6 (3.9) | 41.2 (3.8) | 39.4 (4.1) | 39.3 (4.4) | | 75.8 | 74.1 |
| Creatinine (micromol/L) | 96.5 [25.5] | 93.0 [19.0] | 103.0 [28.0] | 98.0 [22.5] | 112.0 [38.8] | 105.0 [31.0] | | 60.0 | 66.1 |
| eGFR (mL/min/1.73 m^2^) | 70.2 (18.0) | 74.0 (14.6) | 62.5 (16.4) | 66.1 (14.9) | 55.0 (16.0) | 59.4 (16.1) | | 61.4 | 75.5 |
| Sodium (mmol/L) | 139.0 (8.0) | 139.7 (7.5) | 139.0 (8.1) | 139.5 (8.1) | 138.8 (8.9) | 138.9 (9.6) | | 64.5 | 68.0 |
| Potassium (mmol/L) | 4.4 (0.8) | 4.4 (1.1) | 4.6 (4.9) | 4.4 (1.9) | 4.5 (3.4) | 4.4 (2.8) | | 64.5 | 68.0 |
| Total WBC count (10^9 / L) | 8.0 (2.8) | 7.1 (2.2) | 7.7 (2.7) | 7.2 (2.5) | 7.7 (3.2) | 7.4 (2.6) | | 72.9 | 73.9 |
| **Comorbidity (%) §** |  |  |  |  |  |  | |  |  |
| Atrial fibrillation | 6.5 | 0.9 | 8.8 | 2.4 | 11.1 | 4.6 | | n/a | n/a |
| COPD | 22.4 | 10.2 | 25.4 | 13.8 | 27.7 | 18.1 | | n/a | n/a |
| Diabetes mellitus | 5.5 | 1.6 | 5.3 | 2.3 | 3.7 | 2.4 | | n/a | n/a |
| Myocardial infarction | 5.3 | 0.9 | 4.2 | 1.3 | 3.8 | 1.5 | | n/a | n/a |
| Hypertension | 68.5 | 44.8 | 78.8 | 60.2 | 83.4 | 65.4 | | 15.1 | 31.1 |
| Obesity | 19.5 | 11.4 | 13.6 | 7.9 | 6.8 | 4.3 | | 55.7 | 58.6 |
| **Medication use (%) ¤** |  |  |  |  |  |  | |  |  |
| Blood pressure lowering medication | 37.3 | 15.9 | 48.7 | 26.4 | 59.0 | 34.9 | | n/a | n/a |
| Lipid regulating drugs | 29.6 | 13.6 | 26.2 | 17.2 | 11.5 | 10.7 | | n/a | n/a |

*assessed by index of multiple deprivation † measurement closest to and within 3 years before baseline. § denotes prior medical history of given comorbidity 3 years before baseline. SD = Standard Deviation; LDL = Low Density Lipoprotein; HDL = High Density Lipoprotein; SBP = Systolic Blood Pressure; DBP = Diastolic Blood Pressure; total WBC count = total White Blood Cell count; eGFR = estimated Glomerular Filtration Rate; COPD = Chronic Obstructive Pulmonary Disease.

**Supplementary Table 3 - Complete case baseline characteristics stratified by age in women**

|  | **55 – 64 years** | | **65 – 74 years** | | **> 75 years** | | **% missing HF subjects** | **% missing**  **non HF subjects** |
| --- | --- | --- | --- | --- | --- | --- | --- | --- |
|  | HF subjects | Non HF subjects | HF subjects | Non HF subjects | HF subjects | Non HF subjects |  |  |
| Number of patients | 2878 | 254486 | 6624 | 94568 | 15171 | 93315 | 24673 | 442369 |
| **Demographics** |  |  |  |  |  |  |  |  |
| Ethnicity (% Caucasian) | 95.0 | 94.5 | 96.7 | 96.0 | 98.8 | 98.1 | 19.9 | 38.9 |
| Most deprived fifth (%)* | 29.4 | 16.3 | 25.3 | 18.5 | 22.1 | 19.0 | 0.24 | 0.36 |
| **Lifestyle (%) †** |  |  |  |  |  |  |  |  |
| Smoking |  |  |  |  |  |  | 78.1 | 62.4 |
| Current Smoking | 34.8 | 22.0 | 22.3 | 13.9 | 8.2 | 7.1 | n/a | n/a |
| Ex-smoker | 25.7 | 21.2 | 26.1 | 22.9 | 23.1 | 21.4 | n/a | n/a |
| Never smoked | 39.6 | 56.8 | 51.7 | 63.2 | 68.7 | 71.6 | n/a | n/a |
| Sedentary lifestyle | 62.6 | 41.8 | 68.0 | 51.3 | 78.0 | 69.8 | 82.1 | 78.4 |
| **Clinical measures in mean (sd)**  **or median [IQR] †** |  |  |  |  |  |  |  |  |
| Body Mass Index (kg/m^2^) | 31.3 (7.7) | 27.8 (5.8) | 29.0 (6.3) | 27.0 (5.3) | 26.2 (5.2) | 25.1 (4.8) | 61.0 | 54.6 |
| Total cholesterol (mmol/L) | 5.6 (1.2) | 5.8 (1.1) | 5.7 (1.2) | 5.8 (1.2) | 5.7 (1.3) | 5.6 (1.2) | 77.0 | 72.8 |
| Triglycerides (mmol /L) | 2.0 (1.1) | 1.6 (1.0) | 2.0 (1.2) | 1.7 (0.9) | 1.8 (1.1) | 1.6 (0.9) | 86.2 | 80.0 |
| LDL cholesterol (mmol/L) | 3.4 (1.1) | 3.5 (1.0) | 3.5 (1.1) | 3.5 (1.1) | 3.4 (1.2) | 3.3 (1.2) | 90.0 | 83.3 |
| HDL cholesterol (mmol/L) | 1.5 (0.5) | 1.6 (0.5) | 1.5 (0.5) | 1.6 (0.5) | 1.5 (0.5) | 1.6 (0.5) | 89.4 | 82.0 |
| SBP (mmHg) | 145.2 (19.8) | 136.7 (17.7) | 151.5 (20.1) | 148.2 (18.9) | 152.9 (21.0) | 150.2 (20.8) | 23.1 | 25.9 |
| DBP (mmHg) | 83.2 (9.9) | 81.5 (9.3) | 82.6 (9.9) | 82.6 (9.3) | 81.4 (9.9) | 80.8 (10.0) | 23.1 | 25.9 |
| Heamoglobin (g/dL) | 13.3 (1.6) | 13.5 (1.1) | 13.1 (1.5) | 13.3 (1.3) | 12.7 (1.5) | 12.7 (1.5) | 64.0 | 65.7 |
| Platelets (10^9/L) | 265.8 [94.0] | 270.0 [82.0] | 259.0 [93.0] | 263.0 [87.5] | 256.0 [95.8] | 264.0 [96.5] | 69.7 | 69.0 |
| Albumin (g/L) | 40.4 (3.9) | 42.0 (3.5) | 40.2 (3.8) | 40.9 (3.7) | 39. (4.2) | 39.0 (4.4) | 75.8 | 73.1 |
| Creatinine (micromol/L) | 81.0 [23.0] | 77.0 [16.5] | 87.0 [25.5] | 81.5 [20.0] | 94.0 [32.5] | 87.0 [27.0] | 60.0 | 65.2 |
| eGFR (mL/min/1.73 m^2^) | 64.2 (17.8) | 68.9 (14.3) | 56.5 (15.6) | 61.1 (14.5) | 50.5 (15.1) | 55.0 (15.3) | 61.7 | 74.1 |
| Sodium (mmol/L) | 139.3 (7.4) | 139.9 (6.9) | 138.9 (9.1) | 139.4 (9.5) | 138.2 (9.4) | 138.3 (10.1) | 63.9 | 66.7 |
| Potassium (mmol/L) | 4.3 (0.6) | 4.3 (2.4) | 4.5 (8.2) | 4.4 (2.5) | 4.3 (1.2) | 4.3 (2.7) | 63.9 | 66.7 |
| Total WBC count (10^9 / L) | 7.8 (2.5) | 6.7 (2.1) | 7.6 (2.4) | 7.0 (2.3) | 7.6 (2.7) | 7.3 (2.5) | 69.6 | 69.0 |
| **Comorbidity (%) §** |  |  |  |  |  |  |  |  |
| Atrial fibrillation | 5.4 | 0.4 | 7.7 | 1.4 | 10.9 | 3.7 | n/a | n/a |
| COPD | 29.4 | 13.1 | 27.4 | 14.1 | 24.6 | 15.2 | n/a | n/a |
| Diabetes mellitus | 6.5 | 1.1 | 4.9 | 1.7 | 3.4 | 1.9 | n/a | n/a |
| Myocardial infarction | 2.6 | 0.2 | 2.6 | 0.5 | 2.3 | 0.9 | n/a | n/a |
| Hypertension | 72.6 | 46.6 | 81.5 | 63.3 | 86.8 | 70.4 | 11.5 | 21.9 |
| Obesity | 26.4 | 14.5 | 17.2 | 10.4 | 7.4 | 4.8 | 61.0 | 54.6 |
| **Medication use (%) ¤** |  |  |  |  |  |  |  |  |
| Blood pressure lowering medication | 47.2 | 18.9 | 58.6 | 31.8 | 69.1 | 44.2 | n/a | n/a |
| Lipid regulating drugs | 22.9 | 9.4 | 21.6 | 14.3 | 8.8 | 8.7 | n/a | n/a |

*assessed by index of multiple deprivation † measurement closest to and within 3 years before baseline. § denotes prior medical history of given comorbidity 3 years before baseline. SD = Standard Deviation; LDL = Low Density Lipoprotein; HDL = High Density Lipoprotein; SBP = Systolic Blood Pressure; DBP = Diastolic Blood Pressure; total WBC count = total White Blood Cell count; eGFR = estimated Glomerular Filtration Rate; COPD = Chronic Obstructive Pulmonary Disease.

**Supplementary Table 4 - Complete case analysis for risk factors associated with incident heart failure stratified by age in men**

|  | **55 – 64 years** | **65 – 74 years** | **> 75 years** | **Total** |
| --- | --- | --- | --- | --- |
| n (events) | 25,932 (478) | 5,569 (498) | 2,765 (346) | 34,266 (1,322) |
| **Risk Factors** | **HR (95% CI)** | **HR (95% CI)** | **HR (95% CI)** | **HR (95% CI)** |
| Age (years) | 1.05 (1.06; 1.15) | 1.09 (1.03; 1.16) | 1.08 (1.03; 1.12) | 1.08 (1.05; 1.11) |
| Most deprived fifth † | 1.09 (1.21; 2.12) | 1.65 (1.13; 2.42) | 1.40 (0.91; 2.15) | 1.34 (1.07; 1.67) |
| Ex-smokers | 1.27 (0.77: 1.67) | 0.77 (0.53; 1.13) | 0.95 (0.64; 1.41) | 0.98 (0.78; 1.23) |
| Current-smokers | 1.67 (0.71; 1.67) | 1.17 (0.69; 2.01) | 1.50 (0.75; 2.98) | 1.45 (1.09; 1.94) |
| Sedentary lifestyle | 1.11 (1.13; 2.53) | 1.31 (0.92; 1.87) | 1.78 (1.15; 2.74) | 1.34 (1.09; 1.64) |
| Body Mass Index (kg/m^2^) | 1.06 (1.01; 1.05) | 1.01 (0.97; 1.05) | 1.04 (0.99; 1.09) | 1.05 (1.02; 1.07) |
| SBP (per 20 mm/hg) | 0.85 (0.85; 1.26) | 1.06 (0.85; 1.32) | 0.82 (0.64; 1.06) | 0.90 (0.79; 1.04) |
| DBP (per 10 mm/hg) | 0.86 (0.76; 1.02) | 0.82 (0.66; 1.01) | 0.97 (0.75; 1.24) | 0.87 (0.76; 0.99) |
| Haemoglobin (g/dL) | 0.86 (0.91; 1.01) | 0.92 (0.82; 1.04) | 0.91 (0.79; 1.04) | 0.90 (0.84; 0.97) |
| Platelets (per 10 10^9/L) | 0.98 (0.96; 1.00) | 0.98 (0.95; 1.01) | 0.97 (0.94; 1.00) | 0.98 (0.96; 0.99) |
| Total WBC count (10^9 / L) | 1.14 (1.06; 1.16) | 1.01 (0.92; 1.10) | 1.03 (0.94; 1.14) | 1.07 (1.03; 1.12) |
| Total cholesterol (mmol/L) | 0.99 (0.87; 1.12) | 0.94 (0.78; 1.13) | 0.96 (0.78; 1.19) | 0.97 (0.87; 1.08) |
| Triglycerides (mmol /L) | 0.87 (0.90; 1.13) | 1.17 (0.97; 1.42) | 0.91 (0.69; 1.19) | 0.95 (0.86; 1.05) |
| Albumin (g/L) | 0.95 (0.92; 0.99) | 0.92 (0.88; 0.97) | 0.99 (0.94; 1.05) | 0.95 (0.93; 0.98) |
| Creatinine (per 30 µmol/L) | 1.13 (1.03; 1.17) | 1.04 (0.99; 1.09) | 1.23 (1.06; 1.43) | 1.05 (1.02; 1.08) |
| Diabetes | 0.72 (0.37; 1.38) | 1.27 (0.70; 2.32) | 1.31 (0.67; 2.56) | 1.01 (0.70; 1.44) |
| Atrial fibrillation | 2.47 (1.32; 4.60) | 2.59 (1.54; 4.36) | 1.93 (1.13; 3.29) | 2.34 (1.90; 4.24) |
| COPD | 1.87 (1.32; 2.65) | 1.23 (0.80;1.90) | 1.20 (0.76; 1.88) | 1.43 (1.14; 1.79) |

Further adjusted for ethnicity, blood pressure lowering medication and lipid regulating drugs. † Assessed by index of multiple deprivation, HR (95% CI) = Hazard Ratio (95% Confidence Interval), SBP = Systolic Blood Pressure, DBP = Diastolic Blood Pressure, total WBC count = total White Blood Cell count. Hazard ratios were considered statistically significant if p-value < 0.001 (Bonferroni corrected threshold).

**Supplementary Table 5 - Complete case analysis for risk factors associated with incident heart failure stratified by age in women**

|  | **55 – 64 years** | **65 – 74 years** | **> 75 years** | **Total** |
| --- | --- | --- | --- | --- |
| n (events) | 26,036 (227) | 5,846 (325) | 4078 (423) | 35,960 (984) |
| **Risk Factors** | **HR (95% CI)** | **HR (95% CI)** | **HR (95% CI)** | **HR (95% CI)** |
| Age (years) | 1.09 (1.02; 1.16) | 1.08 (1.00; 1.18) | 1.08 (1.04; 1.13) | 1.09 (1.06; 1.12) |
| Most deprived fifth † | 1.17 (0.70; 1.95) | 1.60 (0.96; 2.64) | 1.26 (0.84; 1.90) | 1.34 (1.04; 1.76) |
| Ex-smokers | 0.79 (0.44: 1.43) | 1.76 (1.00; 3.09) | 1.38 (0.95; 2.02) | 1.25 (0.95; 1.64) |
| Current-smokers | 0.99 (0.54; 1.82) | 2.28 (1.13; 4.60) | 1.48 (0.71; 3.11) | 1.50 (1.03; 2.19) |
| Sedentary lifestyle | 1.63 (1.00; 2.68) | 1.35 (0.79; 2.30) | 1.39 (0.90; 2.15) | 1.42 (1.08; 1.87) |
| Body Mass Index (kg/m^2^) | 1.02 (0.98; 1.05) | 1.04 (0.99; 1.09) | 1.00 (0.97; 1.04) | 1.02 (1.00; 1.04) |
| SBP (per 20 mm/hg) | 0.88 (0.63; 1.24) | 0.82 (0.61; 1.12) | 1.00 (0.81; 1.24) | 0.93 (0.80; 1.09) |
| DBP (per 10 mm/hg) | 0.96 (0.71; 1.29) | 0.85 (0.63; 1.14) | 0.98 (0.79; 1.20) | 0.92 (0.80; 1.06) |
| Haemoglobin (g/dL) | 0.94 (0.77; 1.14) | 0.98 (0.79; 1.22) | 0.94 (0.82; 1.09) | 0.93 (0.84; 1.03) |
| Platelets (per 10 10^9/L) | 1.00 (0.96; 1.03) | 0.96 (0.93; 1.00) | 0.98 (0.96; 1.01) | 0.98 (0.96; 1.00) |
| Total WBC count (10^9 / L) | 1.08 (1.00; 1.16) | 0.97 (0.85; 1.12) | 1.05 (0.99; 1.11) | 1.04 (1.00; 1.09) |
| Total cholesterol (mmol/L) | 1.10 (0.88; 1.38) | 0.91 (0.72; 1.15) | 0.96 (0.81; 1.14) | 0.99 (0.89; 1.11) |
| Triglycerides (mmol /L) | 1.02 (0.84; 1.25) | 1.16 (0.90; 1.50) | 0.90 (0.68; 1.19) | 1.01 (0.88; 1.15) |
| Albumin (g/L) | 0.90 (0.84; 0.97) | 0.96 (0.90; 1.02) | 0.97 (0.92; 1.03) | 0.95 (0.94; 0.99) |
| Creatinine (per 30 µmol/L) | 1.06 (0.93; 1.21) | 0.99 (0.72; 1.36) | 1.07 (0.92; 1.24) | 1.04 (0.94; 1.16) |
| Diabetes | 2.76 (1.46; 5.24) | 2.59 (1.17; 5.75) | 1.43 (0.66; 3.11) | 2.28 (1.52; 3.41) |
| Atrial fibrillation | 6.09 (2.52; 14.73) | 5.94 (2.85; 12.33) | 1.34 (0.71; 2.54) | 2.50 (1.66; 3.78) |
| COPD | 2.31 (1.43; 3.75) | 4.15 (2.55; 6.75) | 1.85 (1.21; 2.81) | 2.39 (1.84; 3.09) |

Further adjusted for ethnicity, blood pressure lowering medication and lipid regulating drugs. † Assessed by index of multiple deprivation, HR (95% CI) = Hazard Ratio (95% Confidence Interval), SBP = Systolic Blood Pressure, DBP = Diastolic Blood Pressure, total WBC count = total White Blood Cell count. Hazard ratios were considered statistically significant if p-value < 0.001 (Bonferroni corrected threshold).

**Supplementary Table 6 - Evaluation of heterogeneity at practice level for the association of risk factors with heart failure stratified by age in men**

|  | **55 – 64 years** | **65 – 74 years** | **> 75 years** |
| --- | --- | --- | --- |
| n (events) | 257,698 (5,408) | 88,416 (8,047) | 58,531 (9,859) |
| **Risk Factors** | **HR (95% CI)*** | **HR (95% CI)*** | **HR (95% CI)*** |
| Age (years) | 1.10 (1.09; 1.11) | 1.08 (1.08; 1.09) | 1.07 (1.06; 1.07) |
| Most deprived fifth † | 1.41 (1.31; 1.51) | 1.27 (1.19; 1.35) | 1.08 (1.02; 1.15) |
| Ex-smokers | 1.08 (0.96; 1.21) | 1.03 (0.97; 1.10) | 1.00 (0.94; 1.06) |
| Current-smokers | 1.27 (1.15; 1.41) | 1.15 (1.07; 1.23) | 1.06 (0.96; 1.16) |
| Sedentary lifestyle | 1.06 (0.99; 1.14) | 1.10 (1.04; 1.17) | 1.08 (1.01; 1.15) |
| Body Mass Index (kg/m^2^) | 1.02 (1.01; 1.03) | 1.02 (1.02; 1.03) | 1.01 (1.01; 1.02) |
| SBP (per 20 mm/hg) | 1.03 (0.98; 1.08) | 0.99 (0.96; 1.02) | 0.96 (0.93; 0.99) |
| DBP (per 10 mm/hg) | 0.91 (0.87; 0.95) | 0.90 (0.87; 0.92) | 0.97 (0.94; 0.99) |
| Haemoglobin (g/dL) | 0.96 (0.92; 1.01) | 0.96 (0.95; 0.98) | 0.97 (0.95; 0.99) |
| Platelets (per 10 10^9/L) | 1.00 (0.99; 1.00) | 1.00 (0.99; 1.00) | 1.00 (0.99; 1.00) |
| Total WBC count (10^9 / L) | 1.03 (1.02; 1.05) | 1.02 (1.00; 1.03) | 1.01 (1.00; 1.02) |
| Total cholesterol (mmol/L) | 0.99 (0.95; 1.03) | 0.98 (0.95; 1.01) | 1.02 (0.99; 1.05) |
| Triglycerides (mmol /L) | 1.00 (0.96; 1.03) | 0.99 (0.96; 1.02) | 0.99 (0.96; 1.02) |
| Albumin (g/L) | 0.99 (0.97; 1.00) | 0.99 (0.98; 1.00) | 1.00 (0.99; 1.01) |
| Creatinine (per 30 µmol/L) | 1.03 (1.01; 1.05) | 1.03; 1.01; 1.05) | 1.04 (1.02; 1.06) |
| Diabetes | 1.80 (1.59; 2.04) | 1.69 (1.53; 1.87) | 1.44 (1.30; 1.60) |
| Atrial fibrillation | 4.09 (3.66; 4.58) | 2.52 (2.33; 2.73) | 2.16 (2.02; 2.30) |
| COPD | 1.94 (1.81; 2.07) | 1.82; 1.73; 1.92) | 1.69 (1.62; 1.77) |

*Estimates of random effects accounting for practice level heterogeneity, further adjusted for ethnicity, blood pressure lowering medication and lipid regulating drugs. † Assessed by index of multiple deprivation, HR (95% CI) = Hazard Ratio (95% Confidence Interval), SBP = Systolic Blood Pressure, DBP = Diastolic Blood Pressure, total WBC count = total White Blood Cell count. Hazard ratios were considered statistically significant if p-value < 0.001 (Bonferroni corrected threshold).

**Supplementary Table 7 - Evaluation of heterogeneity at practice level for the association of risk factors with heart failure stratified by age in women**

|  | **55 – 64 years** | **65 – 74 years** | **> 75 years** |
| --- | --- | --- | --- |
| n (events) | 257,364 (2,878) | 101,192 (6,624) | 108,486 (15,171) |
| **Risk Factors** | **HR (95% CI)*** | **HR (95% CI)*** | **HR (95% CI)*** |
| Age (years) | 1.14 (1.12; 1.15) | 1.11 (1.10; 1.12) | 1.06 (1.06; 1.07) |
| Most deprived fifth † | 1.46 (1.33; 1.60) | 1.27 (1.19; 1.36) | 1.12 (1.07; 1.18) |
| Ex-smokers | 1.14 (0.97; 1.34) | 1.05 (0.97; 1.14) | 1.03 (0.98; 1.09) |
| Current-smokers | 1.34 (1.19; 1.51) | 1.21 (1.10; 1.32) | 1.09 (0.99; 1.20) |
| Sedentary lifestyle | 1.09 (1.00; 1.19) | 1.08 (1.01; 1.16) | 1.08 (1.01; 1.14) |
| Body Mass Index (kg/m^2^) | 1.03 (1.03; 1.04) | 1.02 (1.02; 1.03) | 1.01 (1.01; 1.01) |
| SBP (per 20 mm/hg) | 1.11 (1.04; 1.17) | 1.03 (1.00; 1.07) | 0.97 (0.95; 0.99) |
| DBP (per 10 mm/hg) | 0.87 (0.82; 0.92) | 0.91 (0.88; 0.94) | 0.98 (0.96; 1.01) |
| Haemoglobin (g/dL) | 0.95 (0.90; 1.01) | 0.96 (0.93; 0.98) | 0.97 (0.95; 0.98) |
| Platelets (per 10 10^9/L) | 1.00 (0.99; 1.00) | 1.00 (0.99; 1.00) | 1.00 (0.99; 1.00) |
| Total WBC count (10^9 / L) | 1.04 (1.02; 1.06) | 1.02 (1.01; 1.04) | 1.01 (1.01; 1.02) |
| Total cholesterol (mmol/L) | 0.97 (0.92; 1.02) | 0.98 (0.94; 1.01) | 1.01 (0.98; 1.03) |
| Triglycerides (mmol /L) | 1.00 (0.95; 1.05) | 1.01 (0.96; 1.05) | 1.00 (0.97; 1.02) |
| Albumin (g/L) | 0.98 (0.97; 1.00) | 0.99 (0.98; 1.00) | 1.00 (0.99; 1.00) |
| Creatinine (per 30 µmol/L) | 1.06 (1.03; 1.10) | 1.06 (1.02; 1.11) | 1.06 (1.04; 1.08) |
| Diabetes | 2.71 (2.31; 3.18) | 1.89 (1.68; 2.13) | 1.66 (1.52; 1.82) |
| Atrial fibrillation | 6.90 (5.82; 8.19) | 3.51 (3.20; 3.85) | 2.70 (2.56; 2.84) |
| COPD | 2.11 (1.94; 2.29) | 1.94 (1.83; 2.05) | 1.65 (1.59; 1.72) |

* Estimates of random effects accounting for practice level heterogeneity, further adjusted for ethnicity, blood pressure lowering medication and lipid regulating drugs. † Assessed by index of multiple deprivation, HR (95% CI) = Hazard Ratio (95% Confidence Interval), SBP = Systolic Blood Pressure, DBP = Diastolic Blood Pressure, total WBC count = total White Blood Cell count. Hazard ratios were considered statistically significant if p-value < 0.001 (Bonferroni corrected threshold).

**Supplementary Table 8 - Associations of risk factors with incident heart failure stratified by age and endpoints from different sources of EHR in men**

|  | **55 – 64 years** |  | **65 – 74 years** |  | **> 75 years** |  |
| --- | --- | --- | --- | --- | --- | --- |
|  | **CPRD** | **HES** | **CPRD** | **HES** | **CPRD** | **HES** |
| n (events) | 254,731 (2,441) | 255,257 (2,967) | 84,150 (3,781) | 84,635 (4,266) | 53,146 (4,474) | 54,057 (5,385) |
| **Risk Factors** | **HR (95% CI)** | **HR (95% CI)** | **HR (95% CI)** | **HR (95% CI)** | **HR (95% CI)** | **HR (95% CI)** |
| Age (years) | 1.12 (1.11; 1.13) | 1.09 (1.08; 1.11) | 1.09 (1.08; 1.11) | 1.08 (1.07; 1.09) | 1.05 (1.04; 1.06) | 1.08 (1.07; 1.09) |
| Most deprived fifth † | 1.41 (1.28; 1.56) | 1.58 (1.45; 1.72) | 1.24 (1.14; 1.34) | 1.44 (1.33; 1.54) | 1.00 (0.93; 1.08) | 1.15 (1.08; 1.23) |
| Ex-smokers | 1.08 (0.92; 1.27) | 1.07 (0.93; 1.24) | 1.02 (0.92; 1.13) | 1.04 (0.96; 1.14) | 0.99 (0.90; 1.08) | 1.00 (0.93; 1.08) |
| Current-smokers | 1.23 (1.07; 1.42) | 1.31 (1.14; 1.51) | 1.10 (0.97; 1.25) | 1.21 (1.09; 1.34) | 1.05 (0.91; 1.21) | 1.06 (0.94; 1.20) |
| Sedentary lifestyle | 1.09 (0.98; 1.22) | 1.03 (0.93; 1.14) | 1.10 (1.00; 1.22) | 1.12 (1.03; 1.22) | 1.07 (0.97; 1.19) | 1.10 (1.02; 1.19) |
| Body Mass Index (kg/m^2^) | 1.03 (1.02; 1.04) | 1.02 (1.01; 1.03) | 1.03 (1.02; 1.04) | 1.02 (1.01; 1.03) | 1.01 (1.01; 1.02) | 1.01 (1.01; 1.02) |
| SBP (per 20 mm/hg) | 1.01 (0.94; 1.08) | 1.06 (0.99; 1.12) | 0.95 (0.91; 1.00) | 1.04 (1.00; 1.09) | 0.99 (0.95; 1.03) | 0.94 (0.91; 0.98) |
| DBP (per 10 mm/hg) | 0.91 (0.85; 0.97) | 0.90 (0.85; 0.96) | 0.90 (0.86; 0.94) | 0.89 (0.85; 0.93) | 0.97 (0.93; 1.02) | 0.96 (0.92; 0.99) |
| Haemoglobin (g/dL) | 0.97 (0.93; 1.02) | 0.95 (0.89; 1.02) | 0.96 (0.94; 0.99) | 0.96 (0.93; 0.99) | 0.97 (0.94; 1.00) | 0.96 (0.94; 0.98) |
| Platelets (per 10 10^9/L) | 1.00 (0.99; 1.01) | 1.00 (0.99; 1.00) | 1.00 (0.99; 1.00) | 1.00 (0.99; 1.00) | 0.99 (0.99; 1.00) | 1.00 (0.99; 1.00) |
| Total WBC count (10^9 / L) | 1.03 (1.01; 1.06) | 1.03 (1.01; 1.05) | 1.02 (1.00; 1.04) | 1.02 (1.00; 1.03) | 1.02 (1.00; 1.03) | 1.01 (0.99; 1.02) |
| Total cholesterol (mmol/L) | 0.98 (0.92; 1.04) | 1.00 (0.95; 1.06) | 0.99 (0.94; 1.03) | 0.98 (0.94; 1.02) | 1.03 (1.00; 1.07) | 1.01 (0.98; 1.05) |
| Triglycerides (mmol /L) | 1.01 (0.96; 1.05) | 1.00 (0.95; 1.04) | 0.98 (0.93; 1.04) | 1.00 (0.97; 1.04) | 0.99 (0.95; 1.04) | 0.99 (0.95; 1.03) |
| Albumin (g/L) | 0.99 (0.98; 1.00) | 0.99 (0.97; 1.01) | 0.99 (0.97; 1.00) | 0.99 (0.98; 1.00) | 0.99 (0.98; 1.01) | 1.00 (0.99; 1.01) |
| Creatinine (per 30 µmol/L) | 1.03 (0.99; 1.06) | 1.04 (1.01; 1.06) | 1.02; 1.00; 1.05) | 1.03; 1.02; 1.05) | 1.04 (1.01; 1.07) | 1.05 (1.02; 1.08) |
| Diabetes | 1.75 (1.45; 2.10) | 1.88 (1.60; 2.22) | 1.49 (1.27; 1.73) | 1.97 (1.72; 2.24) | 1.27 (1.07; 1.50) | 1.63 (1.42; 1.87) |
| Atrial fibrillation | 4.61 (3.93; 5.39) | 3.83 (3.28; 4.48) | 2.82 (2.52; 3.15) | 2.45 (2.19; 2.75) | 2.22 (2.02; 2.44) | 2.25 (2.07; 2.46) |
| COPD | 2.00 (1.82; 2.20) | 1.90 (1.74; 2.08) | 2.02 (1.88; 2.17) | 1.68 (1.56; 1.80) | 1.91 (1.79; 2.03) | 1.59 (1.50; 1.69) |

* Further adjusted for ethnicity, blood pressure lowering medication and lipid regulating drugs. † Assessed by index of multiple deprivation, EHR = Electronic Health Records, CPRD = Clinical Practice Research Datalink, HES = Hospital Episode Statistics, HR (95% CI) = Hazard Ratio (95% Confidence Interval), SBP = Systolic Blood Pressure, DBP = Diastolic Blood Pressure, total WBC count = total White Blood Cell count. Hazard ratios were considered statistically significant if p-value < 0.001 (Bonferroni corrected threshold).

**Supplementary Table 9 - Associations of risk factors with incident heart failure stratified by age and endpoints from different sources of EHR in women**

|  | **55 – 64 years** |  | **65 – 74 years** |  | **> 75 years** |  |
| --- | --- | --- | --- | --- | --- | --- |
|  | **CPRD** | **HES** | **CPRD** | **HES** | **CPRD** | **HES** |
| n (events) | 255,703 (1,216) | 256,148 (1,662) | 97,522 (2,954) | 98,238 (3,670) | 100,096 (6,781) | 101,705 (8,390) |
| **Risk Factors** | **HR (95% CI)** | **HR (95% CI)** | **HR (95% CI)** | **HR (95% CI)** | **HR (95% CI)** | **HR (95% CI)** |
| Age (years) | 1.16 (1.14; 1.18) | 1.12 (1.10; 1.13) | 1.11 (1.10; 1.13) | 1.11 (1.11; 1.13) | 1.05 (1.05; 1.06) | 1.07 (1.07; 1.08) |
| Most deprived fifth † | 1.53 (1.34; 1.75) | 1.61 (1.44; 1.80) | 1.25 (1.15; 1.37) | 1.36 (1.26; 1.47) | 1.07 (1.01; 1.14) | 1.24 (1.17; 1.30) |
| Ex-smokers | 1.13 (0.88; 1.43) | 1.16 (0.98; 1.36) | 1.02 (0.90; 1.16) | 1.08 (0.98; 1.18) | 1.02 (0.94; 1.11) | 1.03 (0.97; 1.09) |
| Current-smokers | 1.30 (1.04; 1.64) | 1.39 (1.20; 1.61) | 1.17 (1.02; 1.36) | 1.26 (1.11; 1.42) | 1.02 (0.89; 1.18) | 1.15 (1.02; 1.30) |
| Sedentary lifestyle | 1.14 (0.97; 1.32) | 1.07 (0.95; 1.20) | 1.06 (0.94; 1.18) | 1.12 (1.03; 1.21) | 1.08 (1.00; 1.17) | 1.09 (1.01; 1.18) |
| Body Mass Index (kg/m^2^) | 1.03 (1.02; 1.05) | 1.04 (1.03; 1.05) | 1.02 (1.01; 1.03) | 1.02 (1.01; 1.03) | 1.01 (1.00; 1.02) | 1.01 (1.01; 1.02) |
| SBP (per 20 mm/hg) | 1.09 (1.00; 1.19) | 1.13 (1.05; 1.22) | 1.04 (0.99; 1.10) | 1.04 (0.99; 1.09) | 0.98 (0.95; 1.01) | 0.97 (0.94; 1.00) |
| DBP (per 10 mm/hg) | 0.88 (0.80; 0.97) | 0.86 (0.80; 0.93) | 0.92 (0.88; 0.97) | 0.89 (0.85; 0.93) | 1.01 (0.97; 1.04) | 0.96 (0.93; 0.99) |
| Haemoglobin (g/dL) | 0.97 (0.89; 1.05) | 0.93 (0.88; 0.99) | 0.97 (0.93; 1.01) | 0.94 (0.91; 0.97) | 0.97 (0.95; 0.99) | 0.96 (0.93; 0.98) |
| Platelets (per 10 10^9/L) | 1.00 (0.99; 1.01) | 1.00 (0.99; 1.01) | 1.00 (0.99; 1.00) | 1.00 (0.99; 1.01) | 1.00 (0.99; 1.00) | 1.00 (0.99; 1.00) |
| Total WBC count (10^9 / L) | 1.04 (1.01; 1.08) | 1.04 (1.01; 1.07) | 1.03 (1.01; 1.05) | 1.02 (1.00; 1.04) | 1.01 (1.00; 1.03) | 1.02 (1.00; 1.03) |
| Total cholesterol (mmol/L) | 0.94 (0.88; 1.01) | 0.99 (0.92; 1.06) | 0.97 (0.92; 1.03) | 0.98 (0.93; 1.03) | 1.02 (0.99; 1.06) | 1.00 (0.97; 1.03) |
| Triglycerides (mmol /L) | 1.00 (0.92; 1.09) | 1.00 (0.94; 1.06) | 1.01 (0.95; 1.07) | 1.01 (0.96; 1.06) | 0.99 (0.96; 1.03) | 1.00 (0.97; 1.04) |
| Albumin (g/L) | 0.98 (0.97; 1.00) | 0.98 (0.96; 1.00) | 0.99 (0.98; 1.01) | 1.00 (0.98; 1.01) | 0.99 (0.99; 1.00) | 1.00 (0.99; 1.01) |
| Creatinine (per 30 µmol/L) | 1.07 (1.02; 1.12) | 1.06 (1.02; 1.11) | 1.05 (1.00; 1.09) | 1.08 (1.03; 1.14) | 1.06 (1.03; 1.09) | 1.08 (1.05; 1.10) |
| Diabetes | 2.49 (1.94; 3.18) | 2.90 (2.36; 3.57) | 1.60 (1.33; 1.93) | 2.21 (1.90; 2.57) | 1.54 (1.34; 1.77) | 1.90 (1.69; 2.13) |
| Atrial fibrillation | 7.38 (5.76; 9.46) | 7.03 (5.60; 8.82) | 3.83 (3.35; 4.39) | 3.55 (3.13; 4.03) | 2.76 (2.55; 2.98) | 2.95 (2.75; 3.16) |
| COPD | 2.21 (1.95; 2.51) | 2.01 (1.80; 2.24) | 2.08 (1.92; 2.26) | 1.80 (1.67; 1.94) | 1.98 (1.87; 2.09) | 1.47 (1.40; 1.55) |

* Further adjusted for ethnicity, blood pressure lowering medication and lipid regulating drugs. † Assessed by index of multiple deprivation, EHR = Electronic Health Records, CPRD = Clinical Practice Research Datalink, HES = Hospital Episode Statistics, HR (95% CI) = Hazard Ratio (95% Confidence Interval), SBP = Systolic Blood Pressure, DBP = Diastolic Blood Pressure, total WBC count = total White Blood Cell count. Hazard ratios were considered statistically significant if p-value < 0.001 (Bonferroni corrected threshold).
